# Comparative genomic analysis of uropathogenic *Escherichia coli* strains from women with recurrent urinary tract infection

**DOI:** 10.3389/fmicb.2023.1340427

**Published:** 2024-01-24

**Authors:** Marco A. Flores-Oropeza, Sara A. Ochoa, Ariadnna Cruz-Córdova, Rolando Chavez-Tepecano, Eva Martínez-Peñafiel, Daniel Rembao-Bojórquez, Sergio Zavala-Vega, Rigoberto Hernández-Castro, Marcos Flores-Encarnacion, José Arellano-Galindo, Daniel Vélez, Juan Xicohtencatl-Cortes

**Affiliations:** ^1^Posgrado en Ciencias Biomédicas, Instituto de Fisiología Celular, Universidad Nacional Autónoma de México, Mexico City, Mexico; ^2^Laboratorio de Investigación en Bacteriología Intestinal, Unidad de Enfermedades Infecciosas, Hospital Infantil de México Federico Gómez, Mexico City, Mexico; ^3^Ginecología del Hospital Militar Regional Especialidades de Monterrey, Monterrey, Mexico; ^4^Departamento de Patología, Instituto Nacional de Neurología y Neurocirugía, Manuel Velasco Suárez, Mexico City, Mexico; ^5^Laboratorio Clínico y Banco de Sangre, Instituto Nacional de Neurología y Neurocirugía, Manuel Velasco Suárez, Mexico City, Mexico; ^6^Departmento de Ecología de Agentes Patógenos, Hospital General “Dr. Manuel Gea González”, Mexico City, Mexico; ^7^Laboratorio de Microbiología Molecular y Celular, Biomedicina, Facultad de Medicina, BUAP, Puebla, Mexico; ^8^Laboratorio de Virología Clínica y Experimental, Unidad de Investigación en Enfermedades Infecciosas, Hospital Infantil de México Federico Gómez, Mexico City, Mexico; ^9^Hospital Militar de Especialidades de la Mujer y Neonatología, Mexico City, Mexico; ^10^Unidad Médica de Alta Especialidad, Hospital de Ginecología y Obstetricia No. 3 IMSS, Mexico City, Mexico

**Keywords:** UPEC, RUTIs, MDR, WGS, adherence

## Abstract

**Introduction:**

Recurrent urinary tract infections (RUTIs) caused by uropathogenic *Escherichia coli* are costly public health problems impacting patients’ quality of life.

**Aim:**

In this work, a comparative genomics analysis of three clinical RUTI strains isolated from bladder biopsy specimens was performed.

**Materials and methods:**

One hundred seventy-two whole genomes of urinary tract *E. coli* strains were selected from the NCBI database. The search for virulence factors, fitness genes, regions of interest, and genetic elements associated with resistance was manually carried out. The phenotypic characterization of antibiotic resistance, haemolysis, motility, and biofilm formation was performed. Moreover, adherence and invasion assays with human bladder HTB-5 cells, and transmission electron microscopy (TEM) were performed.

**Results:**

The UTI-1_774U and UTI-3_455U/ST1193 strains were associated with the extraintestinal pathotypes, and the UTI-2_245U/ST295 strain was associated with the intestinal pathotype, according to a phylogenetic analysis of 172 *E. coli* urinary strains. The three RUTI strains were of clinical, epidemiological, and zoonotic relevance. Several resistance genes were found within the plasmids of these strains, and a multidrug resistance phenotype was revealed. Other virulence genes associated with CFT073 were not identified in the three RUTI strains (genes for type 1 and P fimbriae, haemolysin *hlyA,* and *sat* toxin). Quantitative adherence analysis showed that UTI-1_774U was significantly (*p* < 0.0001) more adherent to human bladder HTB-5 cells. Quantitative invasion analysis showed that UTI-2_245U was significantly more invasive than the control strains. No haemolysis or biofilm activity was detected in the three RUTI strains. The TEM micrographs showed the presence of short and thin fimbriae only in the UTI-2_245U strain.

**Conclusion:**

The high variability and genetic diversity of the RUTI strains indicate that are a mosaic of virulence, resistance, and fitness genes that could promote recurrence in susceptible patients.

## 1 Introduction

Recurrent urinary tract infections (RUTIs) are a public health problem in Mexico, mainly due to their difficulty in eradicating and the appearance of repeated infectious episodes with disabling signs and symptoms. RUTIs decrease the patient’s quality of life and frequently cause the use of various antibiotic regimens, which can generate bacterial multidrug resistance (Ochoa et al., 2016). UPEC is the main uropathogen recovered from UTIs in vulnerable groups such as children (Contreras-Alvarado et al., 2021), pregnant women (Acuña-Ruíz and Molina-Torres, 2022), women of reproductive age (Ballesteros-Monrreal et al., 2021) and older adults. More than 4 million cases in Mexico have been reported yearly (Secretaría de Salud, 2023). The recurrence rate of UTIs in Mexico has been reported to be 20–23%, depending on age and study groups (Acuña-Ruíz and Molina-Torres, 2022).

Urinary tract infections (UTIs) are an inflammatory response to the colonization and multiplication of a microorganism in any organ of the urinary system. These infections have been associated with dysuria, haematuria, frequent urination, urgency, and occasionally suprapubic pain (Flores-Mireles et al., 2015; Anger et al., 2019; Burnett et al., 2021). The microbiological diagnosis of UTIs is based on the colony counts in a urine culture, and a bacterial concentration of 1 × 10^5^ CFU/mL (colony forming units per milliliter) typically indicates a positive test result according to the Kass-Sandford criteria (Kass, 1956). UTIs occur more frequently in women; the incidence rate of cystitis is 12.6% in women and 3.0% in men in the United States (Wagenlehner et al., 2020). Other data have estimated that more than half of the female population suffer from a UTI episode in their lifetime. Moreover, 25% of women with UTIs experience recurrent infection 3 months after the initial episode (Stamm and Hooton, 1993; Foxman, 2010). Recurrent urinary tract infections (RUTIs) occur when a patient has more than two UTI episodes in 6 months or more than three UTI episodes in a year (Haddad et al., 2020). RUTIs represent a global health problem and can cause chronic infection, poor outcomes, and decreased patient quality of life (Wagenlehner et al., 2018). Recurrent cystitis in women is usually disabling, with 2.78 visits to the physician and 3.45 days of limited activity per year. Many women develop numerous symptoms, and more than 80.3% are treated with various antibiotics. Recurrence prophylaxis is a treatment option; however, the infection was still present in 73% of the patients treated with antibiotic prophylaxis, causing more mental stress in patients (Wagenlehner et al., 2018).

Approximately 80% of all RUTIs are caused by uropathogenic *Escherichia coli* (UPEC) (Thanert et al., 2019). However, the pathophysiology of RUTIs caused by UPEC is a complex process that has not yet been fully characterized. Several virulence factors (VFs) contribute to UPEC pathogenicity, such as characterized fimbriae (Auf, Dr., F1C, S, Type 9, Type 1, and P fimbria), un characterized fimbriae (Type 3 fimbriae), siderophores (enterobactin, aerobactin, and yersiniabactin), toxins (haemolysin, autotransporter secreted toxin, and autotransporter vacuolating toxin), capsule production and variations in lipopolysaccharide (Behzadi, 2020; Khonsari et al., 2021). The presence of metabolic regulators and protectins, increasing serum survival factor (*iss*) and proline permease (*pro*), has been related to the persistence of UPEC (Johnson and Russo, 2018). Multidrug-resistant (MDR) UPEC strains that cause RUTIs are a serious global problem as the antimicrobial treatments necessary to treat them are costly (Brown and Wright, 2016; Habibi and Khameneie, 2016). The discovery of genes encoding extended-spectrum beta-lactamases (ESBL), genes that endow resistance to quinolones, and sulfonamides has contributed vital information to clinical treatment guidelines in Mexico (Alcántar-Curiel et al., 2015). In addition, 5% of RUTIs-associated UPEC strains form intracellular bacterial communities (IBCs) and reactivate quiescent reservoirs (QRs) within the inner layers of the uroepithelium (Mulvey et al., 2001). RUTIs-causing UPEC uses mechanisms such as the production of resistance genes that can lead to treatment failure and the selection of MDR strains that persist in the urinary tract. Moreover, UPEC MDR strains can reactivate once antimicrobial treatment has been completed (Ulett and Schembri, 2016).

Activation of invasins, toxins, siderophores, and metabolic fitness factors contributes to the invasion of uroepithelial cells to form intracellular bacterial communities (IBCs). During this process, UPEC remains within the cells of the transitional urinary epithelium, isolated and protected from antimicrobial treatment and the host’s immune response (Kim et al., 2021). QRs formed by UPEC in the deepest layers of the urinary epithelium can remain for long periods and be reactivated by various signaling systems, a mechanism that has not yet been described in detail; however, these mechanisms can be independent or cooperate to perform a single process (Mulvey et al., 2001).

UPEC strains can acquire mobile genetic elements (plasmids, prophages, or genomic islands) that play a role in horizontal gene transfer (Desvaux et al., 2020). Mobile genetic elements can carry virulence, resistance, and fitness genes that help the bacterium colonize various ecological niches (Baldy-Chudzik et al., 2015). Pathogenicity islands (PAIs) are genomic elements primarily associated with virulence genes that code for resistance determinants, colonization factors, nutrient acquisition, and toxins mediated by specialized secretion systems. PAIs are important elements that distinguish diarrhoeagenic and extraintestinal pathotypes of *E. coli* (Lloyd et al., 2007). Highly virulent strains carrying PAI-I_CFT073_ have been found to carry genes encoding haemolysin, P fimbriae, iron-regulated gene homolog adhesion (*iha*), aerobactin, secreted autotransporter toxin (*sat*), *agn43*, and the “K” capsule widely associated with uropathogenesis (Schneider et al., 2004; Lloyd et al., 2007).

Several treatment options for RUTIs have been proposed, including continuous or postcoital antibiotic prophylaxis, behavioral therapy, treatment with probiotics, treatment with oestrogens, and intravesical instillations of hyaluronate. However, the results have been unsuccessful in their short-term and long-term efficacy (Pigrau and Escolà-Vergé, 2020). In this context, new approaches are needed to design prevention strategies for RUTIs and improve patient quality of life (Behzadi et al., 2023). Bacterial whole-genome sequencing (WGS) is the most recent and up-to-date technology for characterizing the whole genome of UPEC strains, allowing us to understand their genomic context (Voelkerding et al., 2009). This work performed complete genome sequencing of three clinical UPEC strains. The purpose was to conduct a comparison and characterization study on the genomes of UPEC strains isolated from adult women treated at the Military Hospital for Specialties for Women and Neonatology in Mexico City for RUTIs reoccurring over a year. In this study, potential VFs, aptitude factors, and antibiotic resistance were investigated, including the search for elements responsible for the establishment, persistence, and multidrug-resistance (MDRs) of RUTIs. Establishing profiles responsible for recurrence will aid the development of strategies for preventing and treating these infections.

## 2 Materials and methods

### 2.1 Wet lab section

#### 2.1.1 Bacterial strains and sample processing

Three UPEC strains were isolated from the bladder biopsy specimens of three adult women with RUTIs. The patients were treated at the Urology Service of the Military Hospital for Specialties for Women and Neonatology from January 2017 to June 2021. Each patient signed an informed consent letter for the bladder biopsy procedure and urine sample collection.

The bladder biopsy specimens were obtained by the cystoscopy procedure, which was performed by a urologist. They were collected in sterile saline solution for biopsy culture, and collected in formaldehyde for pathological analysis. Bladder biopsy culture was performed at the Intestinal Bacteriology Research Laboratory from the Children’s Hospital of Mexico “Federico Gómez.” The biopsy tissue was mechanically ground in saline solution under sterile conditions and seeded in Petri dishes containing MacConkey agar (BD-BIOXON; Difco BD, 1 Becton Drive, Franklin Lakes, NJ. United States) and 5% sheep blood agar (BD-Difco Becton, Dickinson, and Company, Becton Drive Franklin Lakes, NJ, United States; De Nisco et al., 2019). Characteristic *E. coli* colonies were identified with the MALDI-TOF VITEK MS Microbial Identification System (bioMérieux, 376 Chemin de l’Orme, 69,280 Marcy-l’Étoile, France) at the HIMFG Central Clinical Laboratory. In the Department of Pathology of the National Institute of Neurology and Neurosurgery “Manuel Velasco Suárez,” the pathological analysis of the biopsy specimens was carried out. The tissues were embedded in paraffin, cut with a microtome at 0.5 microns, and stained with hematoxylin-eosin (HE).

Urine samples were collected using the midstream technique and processed for culture in the Central Clinical Laboratory of HIMFG. The samples were seeded in Petri dishes containing cystine-lactose-electrolyte-deficient agar (CLED; BD-BIOXON), MacConkey (BD-BIOXON), and 5% sheep blood agar (BD-Difco) for the identification of classic uropathogens. The microbial count was performed and evaluated according to the Kass-Sandford criterion (Kass, 1956). *E. coli* strains were phenotypically identified using the MALDI-TOF VITEK MS Microbial Identification System (BioMérieux). Subsequently, the strains were propagated on trypticase soy agar plates (TSA; BD-Difco) and preserved in cryovials with trypticase soy broth (TSB; BD-Difco) supplemented with bovine fetal serum (BFS; ATCC, University Boulevard Manassas. VA, United States) at 1% and glycerol (Sigma–Aldrich, Spruce St. Louis, MO, United States) at 20%. The strains were kept at −70°C until use.

#### 2.1.2 Pulsed-field gel electrophoresis assays of UPEC strains

The UPEC strains recovered from each patient’s urine and bladder biopsy were evaluated by Pulsed-field gel electrophoresis (PFGE) to verify their clonal relationship. The PFGE assays were performed according to the protocols established by our working group (Ochoa et al., 2016; Contreras-Alvarado et al., 2021). The UPEC strains were embedded in low-melting-point (LMP) agarose blocks (Promega Corporation, Woods Hollow Road, Madison, WI, United States). The bacterial samples were digested using 20 U of the restriction enzyme Xba1 (New England Biolabs, Ipswich, MA, United States) at 37°C for 20 h. Subsequently, the PFGE was adjusted to run for 24 h at 200 V (7 V/cm) at an angle of 120°C to 14°C, with an initial pulse of 2.16 s and a final pulse of 13.58 s, in a CHEF Mapper system (Bio-Rad Life Science Research, Hercules, CA, United States). The macrorestriction patterns into the agarose gels were stained with GelRed® Nucleic Acid Stain (Biotium, Fremont, CA, United States), visualized with UV light and digitized using a CCD Camera Documenting System BK04S-3 (Biobase, Mexico City, Mexico). PFGE pulsotypes were analyzed with NTSYS pc v2.02j software, and the degree of clonality was evaluated considering the criteria described by Tenover et al. (1995). A lambda ladder PFGE marker (New England Biolabs, Hertfordshire, England, United Kingdom) was used as a molecular weight marker in this assay.

#### 2.1.3 Haemolysis assays

The strains were seeded in Luria–Bertani (LB; DF-Difco) medium and cultured at 37°C overnight under constant agitation at 150 rpm. The suspension was then adjusted to 1.0 OD_600nm_. To assess the presence of haemolysis, the bacteria were plated on agar supplemented with 5% sheep blood and incubated at 37°C for 18 h (Buxton, 2005).

#### 2.1.4 Congo red uptake of UPEC strains

The strains were seeded in LB medium at 37°C overnight under constant agitation at 150 rpm. The suspension was adjusted to 1.0 OD_600nm_ in fresh LB medium. Subsequently, 10 μL was dropped on YESCA agar plates (0.5 g/L yeast extract, 10.0 g/L casaaminoacids, 15.0 g/L bacteriological agar), and 1 mL of 1% Congo red (0.25 g) was added and incubated at 37°C for 72 h to evaluate the uptake of Congo red, an indicator of curli production (Reichhardt et al., 2015).

#### 2.1.5 Motility tests

The strains were grown on LB agar plates (BD-Difco) at 37°C for 24 h. A colony of bacteria was incubated in tryptic soy broth (TSB) medium (BD-Difco) adjusted to an OD_600 nm_ value of 1.0 and then added to a tube with mobility-indole-ornithine medium (MIO; BD-Difco). The positive mobility in the tube was evaluated by the turbidity observed in the medium; negative motility bacteria were limited to the inoculation zone. The confirmatory mobility test was performed in 150 mm x 20 mm diameter Petri dishes containing semisolid mobility medium [0.25% agar (MCD LAB), 1% tryptone (Fluka Analytical), and 0.5% NaCl (J.T. Baker) and 0.005% 2,3,5- triphenyl tetrazolium chloride (TTC; MERCK)]. The medium was incubated with a bacterial colony from the LB plate at 37°C for 18 h. *E. coli* strain W3110 not motile (without halo in mm per 18 h of incubation), UPEC CFT073 motile (halos 24 mm/ 18 h) (Yang et al., 2016), and *Pseudomonas aeruginosa* ATCC 27853 extremely motile (halo 40 mm/ 18 h) (Saeki et al., 2021), were used as controls. The experiments were performed independently in triplicate. The motility obtained by the control strain CFT073 was used as a cut-off value to evaluate the motility of other strains. The UPEC strains were considered mobile when they presented motility halos ≥10 mm and immobile with a halo ≤9 mm.

#### 2.1.6 Quantitative biofilm assays

UPEC strains were grown in TSB medium (BD-Difco) and incubated at 37°C for 24 h under constant agitation at 200 rpm. The 24-well microplates were prepared with 800 μL of TSB medium and 200 μL of the bacterial culture adjusted to an OD_600 nm_ of 1.0. After incubation of the microplate at 37°C for 24 h, three washes were performed with 1 mL of 1x PBS, and the biofilm was fixed with 1 mL of 4% formalin overnight at 4°C. The formalin was gently removed, and the biofilms were dried at room temperature and stained with 1% (w/v) crystal violet for 30 min. The excess dye was removed, the biofilms were washed with PBS 1x three times, and the microplate was allowed to air dry (Gomez et al., 2013). The dye was recovered using 1,000 μL of absolute methanol for 15–20 min in two stages, and the crystal violet retained by the biofilm was spectrophotometrically quantified at 620 nm in a microplate reader (MultiskanTM FC; Thermo Fisher, 81 Wyman Street, Waltham, MA 02451 United States). *E. coli* ATCC 25922, CFT073, and *E. coli* W3110 (lows biofilm former), *E. coli* J96 (moderate biofilm former), and *P. aeruginosa* ATCC 27853 (high biofilm former) (Stepanović et al., 2007; Gomez et al., 2013; Flores-Oropeza, 2017) were used as controls. The profiles of the biofilm-developing strains were categorized as high biofilm formers with ≥0.0180 OD_620nm_ values, moderate biofilm-forming strains with 0.0092–0.0179 OD_620nm_ values, low biofilm-forming strains with values 0.0053–0.0091 OD_620nm_, and non-biofilm-forming strains with values ≤0.0052 OD_620nm_. The experiments were performed independently in triplicate, and the results of all the experiments were plotted and statistically analyzed using GraphPad Prism version 8.0.0 for Windows (GraphPad Software, San Diego, California United States).^1^

#### 2.1.7 HTB-5 cell adherence and invasion assays

The HTB-5 transitional cell carcinoma (TCC) cell line (ATCC, University Boulevard Manassas, VA, United States), which was isolated from the urinary bladder of a 67-year-old female with grade IV TCC, was cultured in culture flasks (Corning) with Eagle Minimum Essential Medium (EMEM; ATCC) and 10% bovine fetal serum (BFS; ATCC) up to 80% confluence. Previously, UPEC strains were grown in 5 mL of TSB medium (BD-DIFCO) to 37°C throughout the night with constant agitation (200 rpm). Next, 1 × 10^5^ HTB-5 cells were seeded in a 24-well microplate in 1 mL of EMEM supplemented with 10% BFS/well. Prior to bacterial infection, the EMEM medium was added to the wells. The cell monolayers were infected at a multiplicity of infection (MOI) of 1:100, and 10 μL of a bacterial suspension adjusted to 1 × 10^7^ bacteria/mL was added and incubated at 37°C for 3 h and 5% CO_2_. Subsequently, bacteria not attached to the cell monolayers were removed with three gentle 1x PBS washes. For quantitative analysis, bacteria adhered to cell monolayers were collected after 1 min incubation with 1 mL PBS/0.1% Triton X-100, and serial dilutions (10^−4^, 10^−5^, and 10^−6^) were grown in Petri plates with LB medium (BD-DIFCO) by the suspended drop method (10 μL) for the counting of CFUs. Finally, for counting invasive bacteria, 1 mL of EMEM medium was added to each well with 100 μg/mL gentamicin and incubated for 1 h. The wells were washed three times with 1x PBS to eliminate dead extracellular bacteria, 0.1% PBS/Triton X-100 solution was added, and the CFUs were counted in direct sample and three dilutions: 10^−1^, 10^−2^, and 10^−3^. Cellular tests were performed independently in triplicate, and CFU/mL values were expressed as the average of the results from the three adherence assays. The averaged adherence and invasion data, along with the standard deviations, were graphed with GraphPad Prism version 8.0.0 for Windows. From the cut-off values of the UPEC strain CFT073, the adherence profiles of the UPEC strains were determined, such as highly adherent (≥2.72 × 10^6^ CFU/mL), moderately adherent (1.18 × 10^6^ – <2.72 × 10^6^ CFU/mL), lowly adherent (8.15 × 10^5^ – <1.18 × 10^6^ CFU/mL), and nonadherent (< 8.15 × 10^5^ CFU/mL). Following the same procedure, the invasive capacity of the UPEC strains was considered highly invasive (≥12.6 CFU/mL), moderately invasive (4.70 – <12.6 CFU/mL), low invasive (1.48 – <4.70 CFU/mL), and non-invasive (<1.48 CFU/mL).

#### 2.1.8 Antibiotic susceptibility assays of UPEC strains

The minimum inhibitory concentration (MIC) was determined by the automated method of the VITEK 2 Systems based on the Therapeutic Microbiological Interpretation Guide. The Clinical and Laboratory Standards Institute (CLSI, 2022) guidelines (section 2.2) and Natural Resistance 2021 were used to determine the antibiotic susceptibility profile using 15 antibiotics belonging to 8 antibiotic categories: β-Lactam combination agents [Ampicillin-sulbactam (AMS), and Piperacillin-tazobactam (TZP)], Cephems 2nd generation: [Cefoxitin (FOX); Cephems 3rd generation: Ceftazidime (CAZ), and Ceftriaxone (CRO)], Cephems 4th generation: [Cefepime (CEF)], Carbapenems: [Doripenem (DOR), Ertapenem (ERT), Meropenem (MEM), and Imipenem (IPM)], Aminoglycosides: [Gentamicin (GM), and Amikacin (AMK)], Fluoroquinolones: [Ciprofloxacin (CIP)], Lipopeptides: [Colistin (COL)], and Glycylcycline: [Tigecycline (TIG)]. The reference strains *E. coli* ATCC 25922 (β-lactamase negative) and *E. coli* ATCC 35218 (ESBL-TEM-1 producer) were used as controls. The methodology for antibiotic susceptibility was performed according to the guidelines of the CLSI (2022) and Ochoa et al. (2016). MDR strains are characterized by having acquired no susceptibility to at least one antibiotic in three or more classes. XDR strains have nonsusceptibility to at least one agent in all performed but two or fewer antibiotic classes (Magiorakos et al., 2012).

#### 2.1.9 Phenotypic determination of ESBL and metallo-beta-lactamase in UPEC strains

Antibiotic disk susceptibility assays with CAZ (30 μg), aztreonam (ATM-30 μg), and CRO (30 μg) were performed for the presumptive identification of ESBL. In addition, antibiotic disks with MEM (10 μg) and IPM (10 μg) were used for metallo-beta-lactamase (MBL) identification, as suggested by CLSI (2022). Both enzyme groups were subjected to synergism assays with double and single disk assays. *Klebsiella pneumoniae* ATCC 700603 (ESBL+), *E. coli* ATCC 25922 (ESBL-), *P. aeruginosa* ATCC 27853 (MBL-), and clinical *P. aeruginosa* 540UC1 (MBL+) were used as control strains (Ochoa et al., 2015, 2016). Also, the phenotypic determination of ESBL production was performed using the VITEK 2 Systems automated method (Therapeutic Microbiological Interpretation Guide, CLSI section 2.2, and Natural Resistance 2021). The phenotypic assays were performed in triplicate.

### 2.2 Dry lab section

#### 2.2.1 Sequencing and assembly of UPEC strains

The UPEC strains recovered from the bladder biopsy specimens were sent to the Sequencing Service of the Center for Genomic Sciences-UNAM. The genome was sequenced with the Illumina HiSeq platform (Illumina, Inc. 5,200 Illumina Way, San Diego, CA 92122, United States) and the Oxford Nanopore MiniION platform (Gosling Building, Edmund Halley Road, Oxford Science Park OX4 4DQ, United Kingdom). Hybrid assembly was performed *de novo* with Unicicler v. v0.4.1.^2^ The assemblies were deposited in the BioProject database under the accession number PRJNA610084.^3^

#### 2.2.2 Annotation and comparison of complete UPEC strain genomes.

The three assembled genomes were annotated with the NCBI Prokaryotic Genome annotation server^4^ using the best-placed reference protein set method. In addition, they were annotated with GeneMarkS-2 + v4.11 (Thibaud-Nissen et al., 2016).^5^

The comparison of the complete genomes was performed using the MAUVE bioinformatics tools LASTZ and Geneious, included in the Geneious Prime 2023.0.3 software.^6^ The loci with nucleotide differences, based on the alignments, were verified with SnapGene®-2023 software.^7^ The hypothetical proteins were analyzed with InterProScan v5.65–97.0 software^8^ (Paysan-Lafosse et al., 2023) to classify them by families and predict their functional domains. The infrequent regions identified during the WGS alignment and related to adhesion were called regions of interest.

#### 2.2.3 Construction of the UPEC strain phylogenetic tree

In the NCBI^9^ and BV-BRC^10^ databases, a search for the genomes of “*E. coli*,” from “Human” OR “*Homo sapiens*,” was performed with the following characteristics: sequence status “Complete,” source of isolation “urine” OR “urinary tract,” and disease “urinary tract infection” OR “UTI” OR “cystitis” OR “pyelonephritis.” The final database was manually curated. In addition, 160 genomes associated with the urinary tract were selected, and 11 genomes were characterized as controls [*E. fergusonii* ATCC 35469 (CU928158), EAEC/STEC 042 (FN554766), EHEC Sakai (CP028307), EIEC CFSAN029787 (CP011416), EPEC XH987 (CP102675), ETEC H10407 (FN649414), K12 MG1655 (U00096), K12 W3110 (CP017979), NMEC S88 (CU928161), *Shigella flexneri* 2a ATCC 29903 (CP026788), and STEC 00–3,076 (CP027584)], through the service of BV-BRC 3.30.19 Bacterial Genome Tree.^11^ For the construction of the phylogenetic tree, the file in Newick format (.nwk) was exported to iTOL v6.8,^12^ for annotation, and finally, the Scalable Vector Graphics file (.svg) was edited in the Inkscape v1.2.2 program.^13^

#### 2.2.4 Identification of virulence, fitness and resistance genes

The search for virulence and resistance genes (antibiotics, heavy metals, and quaternary salts) in chromosomes and plasmids was performed using the VirulenceFinder v2.0 servers,^14^ VRprofile2 v2.0,^15^ OriTFinder 1.1,^16^ VICTORs and PATRIC from the Bacterial and Viral Bioinformatics Resource Center (BV-BCR) 3.28. 9,^17^ ResFinder 4.1^18^ and The Comprehensive Antibiotic Resistance Database.^19^ The results were compared using the CLUSTAL Omega bioinformatics tools v1.2.22 in the Geneious Prime software, UNIPROT 2023^20^ (The UniProt Consortium, 2023) and NCBI.^21^

#### 2.2.5 Identification of phylogenetic groups, genomic Islands and prophages

The phylogenetic group of the UPEC strains was identified using the EzClermont 0.7.0 command tool using the following link: https://ezclermont.hutton.ac.uk/ (Beghain et al., 2018; Clermont et al., 2019). The search for Genomic Islands as elements capable of transferring virulence between strains was carried out using IslandViewer v.4.0^22^ (Bertelli et al., 2017) and VRprofile v.2.0 (see footnote 15, respectively; Wang et al., 2022). The presence of prophages was determined by employing the PHASTER 4.3X platform^23^ (Zhou et al., 2011; Arndt et al., 2016).

#### 2.2.6 Determination of UPEC serotypes, sequence types, and clonal complexes

The serotypes of the UPEC strains were evaluated with the SeroTypeFinder 2.0 server^24^ for the identification of the following antigens: somatic (O) and flagellar (H) (Joensen et al., 2015). The STs of the UPEC strains were determined by allele identification in the sequences of seven “Housekeeping” genes suggested by the University of Oxford’s Multilocus Sequence Type (MLST) web platform. The alleles in the gene sequences *adk* (adenylate kinase), *fumC* (fumarate hydratase), *gyrB* (DNA gyrase), *icd* (isocitrate/isopropyl malate dehydrogenase), *mdh* (malate dehydrogenase), *purA* (adenylosuccinate dehydrogenase), and *recA* (ATP/GTP binding motif) were identified with the platform^25^ (Jolley et al., 2018).

## 3 Results

### 3.1 Phenotypic characterization

#### 3.1.1 Patient clinical-pathological data

UPEC strains were recovered from 3 adult women with RUTIs for at least 1.5 years treated at the Urology Service of the Military Hospital for Specialties for Women and Neonatology, Secretary of National Defense, Mexico City. Bladder biopsy specimens were collected from each patient 1 month after concluding antimicrobial and topical treatment. Patient 1 (P1) was diagnosed with follicular or bullous cystitis and had no history of comorbidities. Patient 2 (P2) presented a diagnosis of interstitial cystitis and suffered a lumbar trauma that led to a bladder emptying disorder; patient 2 also presented type 2 diabetes mellitus. Patient 3 (P3) was diagnosed with moderate stress mixed urinary incontinence and cyclic neutropenia according to clinical data. The clinical strains of RUTIs recovered from the patients were called UTI-1_774U (P1), UTI-2_245U (P2), and UTI-3_455U (P3). The patients received treatment with multiple broad-spectrum antibiotics, such as amoxicillin/clavulanic acid and nitrofurantoin, over the course of infection. In addition, nonantibiotic topical treatment was applied with intravesical instillations of hyaluronate, anticholinergics, and local oestrogens. The clinical data of each patient, including age, antibiotic/nonantibiotic treatment, topical application, recovered strain, and biological evaluation of the clinical sample, are shown in Table 1 and Supplementary Figure S1.

**Table 1:** Clinical description of patients with RUTIs obtained of the military hospital for specialties for women and neonatology in Mexico City, Mexico.

Patient	P1	P2	P3
Strain	UTI-1_774U	UTI-2_245U	UTI-3_455U
Age (sex)	50	30	60
Clinical antecedents	Bullous cystitis	Interstitial cystitis, diabetes, lumbar trauma, and voiding dysfunction	Cyclic neutropenia and urinary incontinence.
Initial treatment date	2017–01	2018–03	2018–12
Disease evolution (years)	3.25	2.0	1.58
Last antibiotic treatment	Amoxicillin/clavulanic acid	ND	Phenazopyridine/nitrofurantoin
Systemic treatment	Intravesical installation of hyaluronate (12 doses)	Intravesical installation of hyaluronate (12 doses)	ND
Prophylaxis, replacement, and psychological treatment	Oral lactobacilli and nitrofurantoin	Anticholinergic and behavior therapy	Oral lactobacilli, topic estrogens, and behavior therapy
Biological sample	Concentrated urine in 1000 CFU/mL and bladder biopsy	Urine culture 100,000 CFU/mL, bladder biopsy	Urine culture 100,000 CFU/mL and bladder biopsy

P1 (Patient 1); P2 (Patient 2); P3 (Patient 3); UTI, Urinary Tract Infection; UPEC, Uropathogenic Escherichia coli; CFU/mL (Colony Forming Unit per milliliter); ND, nondate.

**Figure 1:**
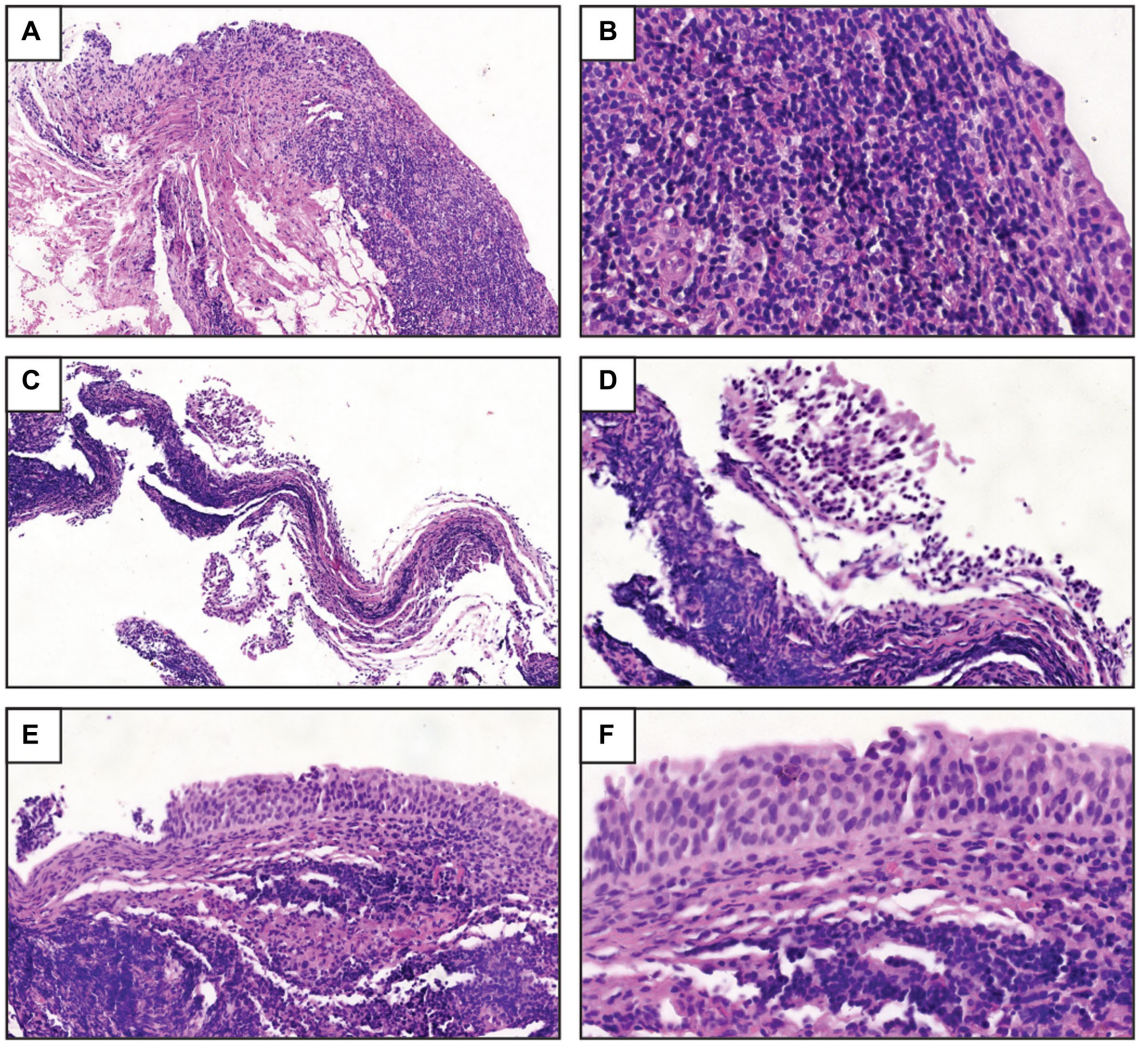
Histopathological study of the biopsy samples. **(A)** UTI-1_774U biopsy sample obtained from women with RUTIs showed a severe inflammatory response and lymphocytic infiltration in the submucosa [hematoxylin-eosin stain (HES), 10X]. **(B)** UTI-1_774U biopsy sample with discrete exocytosis of lymphocytes through the urothelial epithelium (HES, 40X). **(C)** UTI-2_245U biopsy sample with intense chronic inflammation and mucosal detachment (HES, 10X). **(D)** Marked cellularity and inflammatory response in the submucosa (HES, 20X) of UTI-2_245U biopsy sections. **(E)** UTI-3_455U biopsy sample with chronic nonspecific lymphocytic inflammation in the submucosa (HES, 20X). **(F)** UTI-3_455U biopsy sample with focal epithelium detachment (HES, 40X).

The histopathological micrographs of the three-patient bladder biopsy specimens showed a severe inflammatory response (Figure 1). The bladder tissue of P1 showed an intense lymphocytic inflammatory infiltrate in the submucosa with discrete exocytosis of lymphocytes through the urothelial epithelium (Figures 1A,B). The bladder tissue of P2 also showed severe chronic lymphocytic inflammation with total involvement of the mucosa, including mucosal detachment (Figure 1C). Significant inflammatory cellularity was observed in the submucosa (Figure 1D). In the bladder tissue of P3, chronic nonspecific lymphocytic inflammation in the submucosa was demonstrated, and focal detachment of the epithelium was also observed (Figures 1E,F).

#### 3.1.2 Haemolytic activity, adherence, and invasion of UPEC strains

The haemolytic activity of the UPEC strains UTI-1_774U, UTI-2_245U, and UTI-3_455U visualized in agar supplemented with 5–10% sheep blood showed a haemolytic activity similar to that of the negative control *E. coli* strain W3110 (Supplementary Figure S2). Moreover, the genes associated with haemolytic activity in the tested strains are listed in Supplementary Table S1. The UPEC strain CFT073 used as a positive control showed haemolytic activity in a β-haemolysis zone around the inoculation site (Supplementary Figure S2).

**Figure 2:**
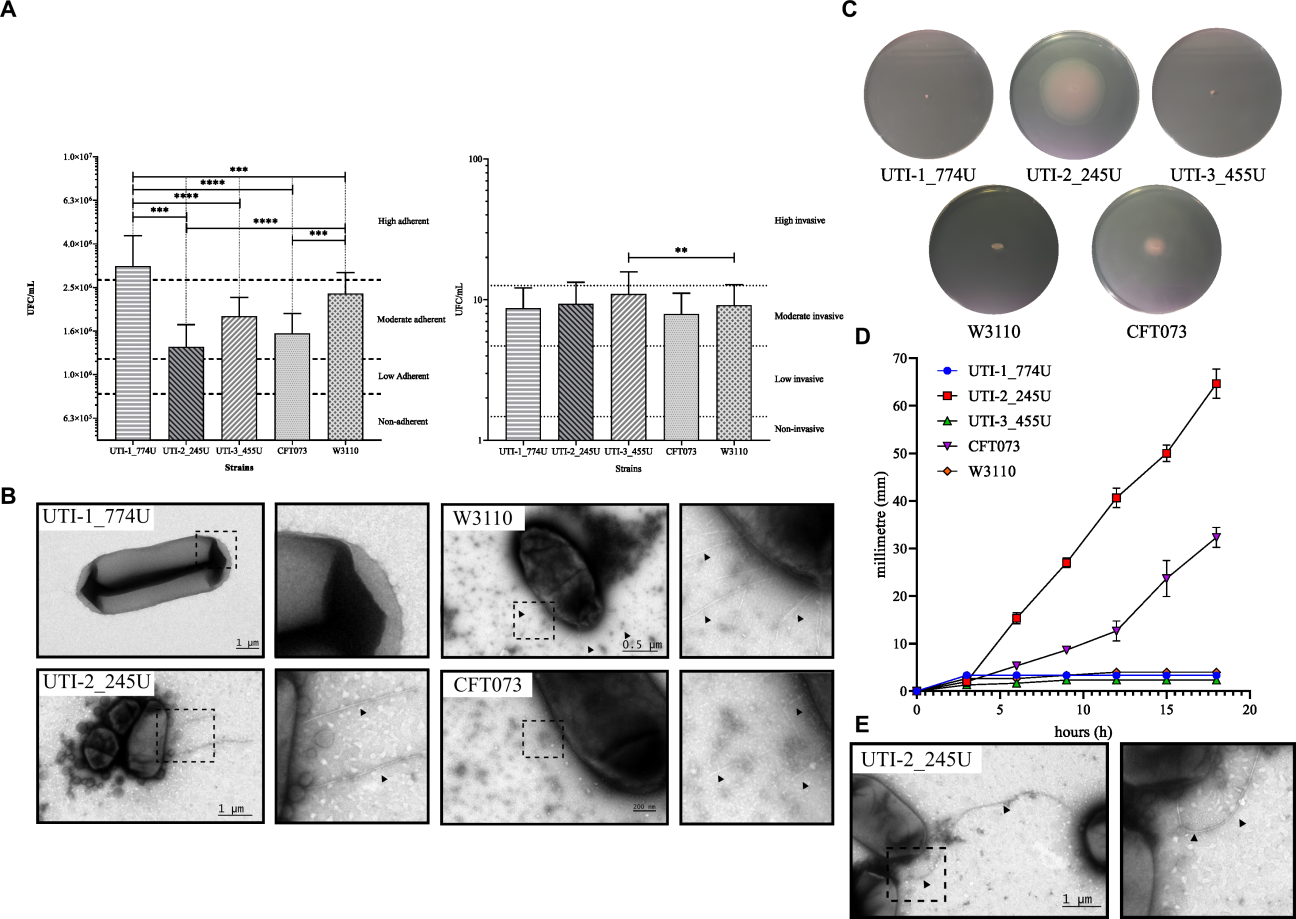
Phenotype assays of RUTI strains. **(A)** Adherence and invasion assays of HTB-5 human bladder cells. RUTI strains are adherent and invasive; the UTI-1_774U strain is highly adherent, while the UTI-3_455U strain is moderate invasive. **(B)** The fimbria of the RUTI strains and the controls are visualized by TEM. **(C)** Mobility assays and mobility plates with tetrazolium chloride show a halo of mobility around the colony. UPEC strains UTI-1_774U and UTI-3_455U are not motile. The mobility speed graph shows that the UTI-2_245U strain is highly mobile. **(D)** Quantitative analysis of the motility by flagella of the three clinical strains. UTI-2_245U strain shown a motility developed. **(E)** TEM micrography showing the flagella in the UTI-2_245U strain.

Quantitative analysis of adherence showed that the UTI-1_774U strain (3.15×10^6^ CFU/mL) was significantly (*p* < 0.0001) highly adherent to HTB-5 cells in comparison with the strains UTI-3_455U (1.85 × 10^6^ CFU/mL), UTI-2_245U (1.34 × 10^6^ CFU/mL), UPEC CFT073 (1.54 × 10^6^ CFU/mL), and *E. coli* W3110 (2.36 × 10^6^ CFU/mL), as shown in Figure 2A. The quantitative analysis of invasion showed that the UTI-3_455U strain (11 CFU/mL) was significantly invasive to HTB-5 cells when compared to the strains UTI-1_774U (8.72 CFU/mL), UTI-2_245U (9.33 CFU/mL), UPEC CFT073 (7.92 CFU/mL), and *E. coli* W3110 (9.15 CFU/mL). In addition, complete genome analysis using the PATRIC and NCBI platforms showed the presence of genes associated with adherence and invasion in the strains (Supplementary Figure S1).

The presence of fimbriae potentially involved in the adhesion and invasion processes under the described conditions was subsequently evaluated by TEM. Negative staining TEM micrographs with 0.75% uranyl acetate showed the presence of short (>1 μm) and thin fimbriae in UTI-2_245U. However, in the micrographs of strains UTI-1_774U and UTI-3_455U, the fimbria were not observed under these growth conditions. The control strains of *E. coli* W3110 and UPEC CFT073 also produced shorter and thinner fimbriae than those of UTI-2_245U (Figure 2). On the other hand, the three recurrent UPEC strains assembled the curli fimbriae when cultured overnight, at 37^ο^C and 20°C, and YESCA medium (data not shown).

#### 3.1.3 Biofilm formation by UPEC strains

The formation of biofilms by the spectrophotometric quantification method of the crystal violet dye retained by the biofilm at 620 nm showed that the UPEC strains UTI-1_774U (OD_620 nm_ = 0.0003), UTI-2_245U (OD_620 nm_ = 0.0019) and UTI -3_455U (OD_620 nm_ = 0.0018) were not biofilm formers when compared with the positive control strains *Pseudomonas aeruginosa* (OD_620 nm_ = 0.0981) and UPEC J96 (OD_620 nm_ = 0.0143) and with the negative control strains UPEC CFT073 (OD_620 nm_ = 0.0034) and *E. coli* ATCC 25922 (OD_620 nm_ = 0.0043) (Supplementary Figure S3). The bioinformatic analysis of the sequenced genomes showed the presence of genes associated with biofilm formation in the UPEC strains. These genes are shown in Supplementary Table S1.

### 3.2 Genotypic characterization

#### 3.2.1 General characteristics of the UPEC genomes

The sequence analysis of the three UPEC strains showed that the genomes were different sizes: 5.19 Mb for UTI-1_774U, 5.01 Mb for UTI-2_245U, and 5.0 Mb for UTI-3_455U. Comparative analysis of the three UPEC genomes using the local pairwise alignment approach showed 99% overall identity between UTI-1_774U and UTI-3_455U, 93% identity between UTI-2_245U and UTI-3_455U, and 88% identity between UTI-1_774U and UTI-2_245U (Supplementary Table S2). Comparative analysis of the whole genomes showed the lack of PAIs in the UTI-1_774U, UTI-2_245U, and UTI-3_455U strains when compared with UPEC CFT073 (Supplementary Figure S4). The whole genome of the UTI-1_774U strain contained 4,954 genes, 160 pseudogenes, and 292 genes that code for hypothetical proteins (Table 2). This strain harbors a cryptic plasmid of 2,113 bp, a nonconjugative mobilization plasmid of 4,072 bp, a conjugative mobilization plasmid of 5,631 bp, and a resistance carrier plasmid of 102,591 bp (Table 2). All data obtained from the bioinformatics databases of the 172 genomes included in this study are displayed in the Excel Supplementary Database.

**Table 2:** General description of the genomes of the three UPEC strains associated to RUTIs.

Strain	Source of DNA	GenBank®	Size (bp)	Genes (Total)	CDSs (Total)	Genes (coding)	Pseudogenes	Hypothetic proteins	Plasmid function
UTI-1_ 774 U	Chromosome	CP049852.1	5,192,928	4,954	4,835	4,675	160	292	
	pEcoUTI1a	CP049856.1	2,113	2	2	1	0	1	Small cryptic
	pEcoUTI1b	CP049855.1	4,072	3	3	3	0	0	Non-conjugative mobilization
	pEcoUTI1c	CP049854.1	5,631	7	7	4	2	1	Conjugative mobilization
	pEcoUTI1d	CP049853.1	102,591	117	117	46	56	15	Resistance
UTI-2_ 245 U	Chromosome	CP049845.1	5,016,451	5,205	5,080	4,927	153	280	
	pEcoUTI2a	CP049851.1	5,119	10	10	5	2	3	Colicin production
	pEcoUTI2b	CP049850.1	5,631	7	7	4	2	1	Conjugative mobilization
	pEcoUTI2c	CP049849.1	6,336	8	8	1	6	1	Resistance
	pEcoUTI2d	CP049848.1	68,488	89	89	67	2	20	Fertility
	pEcoUTI2e	CP049847.1	90,408	107	104	61	16	30	Extrachromosomal-phage P1
	pEcoUTI2f	CP049846.1	109,478	126	126	90	10	26	Resistance
UTI-3_ 455 U	Chromosome	CP049839.1	5,003,672	5,049	4,930	4,770	160	296	
	pEcoUTI3a	CP049843.1	1,822	2	2	2	0	0	Small cryptic
	pEcoUTI3b	CP049842.1	2,113	2	2	1	0	1	Small cryptic
	pEcoUTI3c	CP049841.1	5,630	7	7	6	0	1	Conjugative mobilization
	pEcoUTI3d	CP049844.1	88,163	97	97	78	2	17	Conjugative
	pEcoUTI3e	CP049840.1	91,528	103	103	38	48	17	Resistance

CDS, Coding Sequence; pEcoUTI, plasmid from *Escherichia coli* urinary tract infection strain.

The genome of the UTI-2_245U strain contains 5,205 genes, 153 pseudogenes, and 280 genes encoding hypothetical proteins. In addition, it contains six plasmids, a 5,119 bp colistin production-associated plasmid, a 5,631 bp conjugative mobilization plasmid, a 6,336 bp resistance plasmid, a 68,488 bp fertility plasmid, a phage plasmid from 90,408 bp, and a resistance plasmid from 109,478 bp (Table 2; Excel Supplementary Database). The genome of the UTI-3_455U strain contains 5,049 genes, 160 pseudogenes, and 296 genes encoding hypothetical proteins. Furthermore, it contains five plasmids, two small cryptic plasmids (1,822 bp and 2,113 bp), a 5,630 bp conjugative mobilization plasmid, an 88,163 bp conjugation plasmid, and a resistance plasmid of 91,528 bp (Table 2; Excel Supplementary Database). The plasmids found in the UPEC strains associated with RUTIs, UTI-1_774U, UTI-2_245U, and UTI-3_455U, are shown in the Supplementary Figure S5. The UTI-2_245U genome contains the *cas* I-E operon and two CRISPR arrays with 13 spacers, similar to the genomes of several enterobacteria, mainly the serotypes of other *E. coli* and serogroups of *Shigella* spp. Interestingly, high similarity to the bacteriophage *Myoviridae* sp. ctitt1 was also identified. In contrast, the UTI-1_774U and UTI-3_455U strains did not display CRISPR *Cas* genes; however, four repeat and spacer regions with high sequence similarity to *Enterobacteriaceae* were identified (Supplementary Table S3).

#### 3.2.2 Phylogenetic comparison of UPEC strains

**Figure 3:**
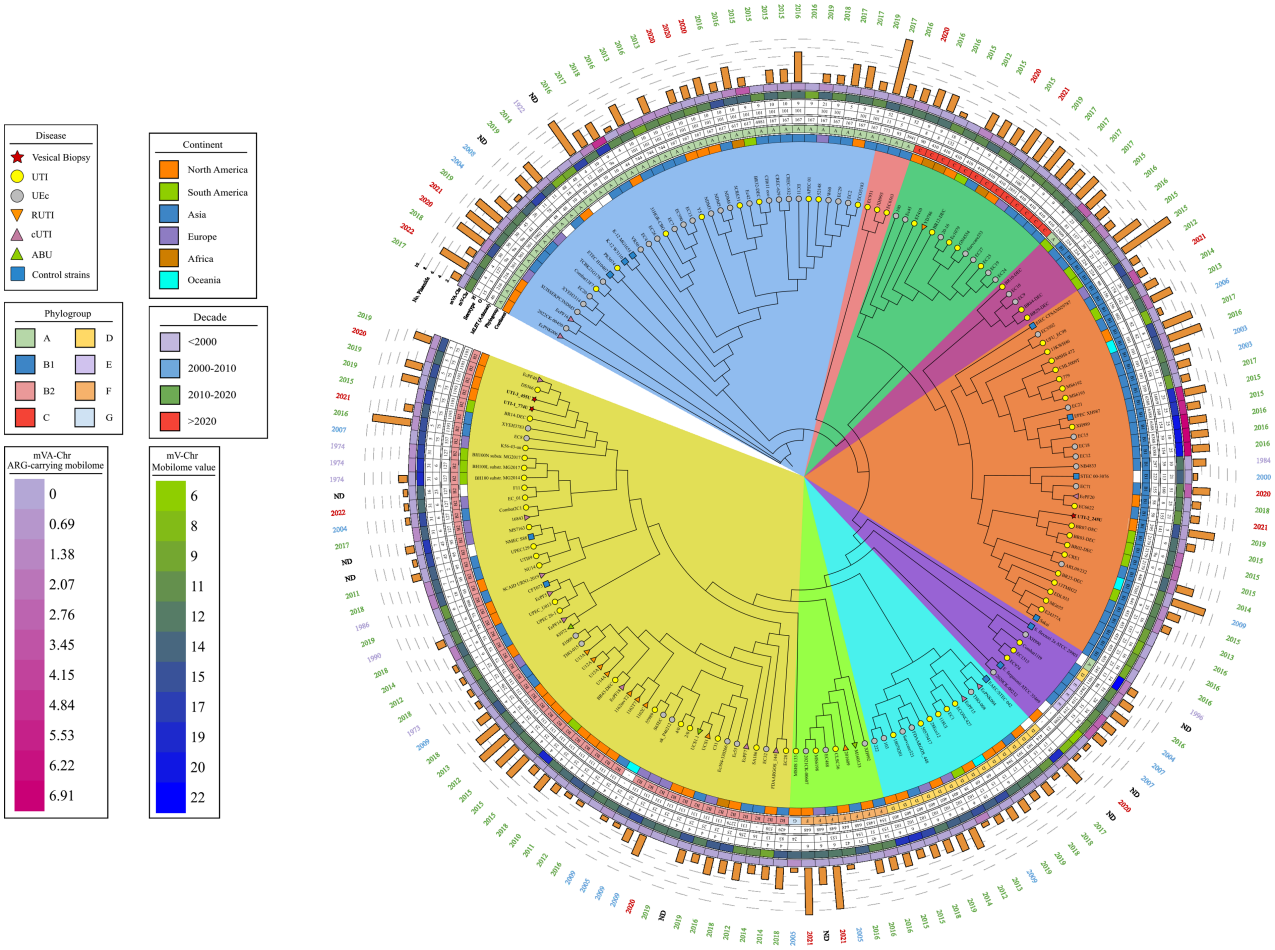
Phylogenetic map of 172 uEC strains and controls. The phylogenetic tree obtained through the BV-BCR 3.30.19 service, Bacterial Genome Tree, is presented. The disease data, phylogroup, continent, date of isolation, and mobilome of the strains are presented, and colored shadows represent the clades obtained. Cluster 1 (blue), cluster 2 (pink), cluster 3 (green), cluster 4 (violet), cluster 5 (orange), cluster 6 (purple), cluster 7 (light blue), cluster 8 (lime green), and cluster 9 (yellow).

The comparative phylogenetic analysis included 157 strains of *E. coli* associated with urinary infection, 11 strains of *E. coli* control strains, and the three isolated RUTIs strains. These strains were distributed into nine well-defined clusters according to their phylogenetic group, STs, serotype, and their origin of isolation (Figure 3). In cluster 1, 33 clinical strains of urinary *E. coli* and three control strains (ETEC H10407, *E. coli* K12 W3110, and *E. coli* K12 MG1655) were distributed. In addition, 52.77% (19/36) of them were isolates mainly of Asian descent, 100% (36/36) belonged to phylogenetic group A, 36.11% (13/36) belonged to ST167, 52.77% (19/36) belonged to serogroup O101, and 30.55% (11/36) contained the H10 flagellar antigen. The predominant serotypes were O101:H10, 30.55% (11/36), and O101:H9, 16.66% (6/36). In cluster 2, three UPEC strains were isolated from Asia, all belonging to phylogenetic group A. Thirteen strains were grouped in cluster 3, and 38.46% (5/13) were from Asia; while 23.07% (3/13) were from North America and Africa. Moreover, 100% (13/13) of these stains belonged to phylogenetic group C, 84.61% (11/13) belonged mainly to ST410, and 76.92% (10/13) had the H9 flagella antigen. The most frequent serotype was O8:H9 in 23.07% (3/13) (Figure 3). Five clinical strains of UPEC were grouped in cluster 4, and 60% (3/5) were of South American origin. Moreover, 80% (4/5) belonged to the phylogenetic group B1, ST224, and flagella antigen H23. The strain EC10 O157:H7 recovered from urine was included in this cluster. Twenty-eight clinical strains of UPEC and four control strains (EIEC CFSAN029787, EPEC XH987, STEC 00–3,076, and *E. coli* Sakai O157:H7) were grouped in cluster 5, 62.5% were of Asian origin and all belonged to phylogenetic group B1. In this cluster, *E. coli* strains from urinary infections with a wide variety of “O” (O23, O9, O160, O:82, O:188, O:154, and O157) and “H” (H25, H:16, H:31, H8, H9, and H21) antigens and ST (ST6388, ST453, ST101, and ST2179) were grouped. In addition, the RUTIs clinical strain UTI-2_245U was grouped, with serotypes O141:H5 and ST295 and phylogenetic group B1. In this clade, strains related to UTIs and urinary *E. coli* were grouped (Figure 3). In cluster 6, five clinical strains of UPEC and two control strains (*Shigella flexneri* 2a ATCC 29903 and *Escherichia fergusonii* ATCC 35469) were distributed. Additionally, 42.85% (3/7) belonged to phylogenetic groups E, ST219, ST2847, serotype O51:H34, and H28. In this cluster, a clinical strain of phylogroup A (O13:H16/ST245) and a clinical strain of phylogroup D (O86:H51/ST1011) were also grouped. In cluster 7, 13 clinical strains of UPEC and one control strain (EAEC_STEC_142) were grouped. In addition, 100% (14/14) belonged to phylogenetic group D, and 50% (7/14) belonged to serogroup O102. Of these strains, 57.14% (8/14) showed a flagellar antigen H6, 42.85% (6/14) were ST405, and 50% (7/14) were of North American origin (Figure 3). Eight clinical strains of UPEC were grouped in cluster 8, and 87.5% (7/8) belonged to phylogroup F, while 50% (4/8) were of North American and Asian origin. Of them, 62.5% (5/8) belonged to ST648, 50% (4/8) belonged to two serogroups (O1 and O153), and 37.5% (3/8) were flagellar antigen H6. In this cluster, one strain belonging to phylogenetic group G showed serogroup O24 and was of North America. Finally, in cluster 9, 52 UPEC strains and a control strain of extraintestinal *E. coli* (NMEC 588) were grouped. Of these strains, 100% (53/53) belonged to phylogenetic group B2, 43.39% (23/53) were associated with serogroup O25, 43.39% (23/53) showed the flagellar antigen H4, 39.62% (21/53) were ST131, 37.73% (20/53) were of North America, and 28.30% (15/53) were of Asian origin. The most frequent serotypes in this cluster were the following: strains 33.73% (20/53) were O25:H4/ST131, 13.20% (7/53) were O75:H5/ST1193, 9.42% (5/53) were O6:H31/ST127, and 7.54% (4/53) were O6:H1/ST73. The two RUTIs strains (UTI-1_774U and UTI-3_455U) were grouped in this cluster and belonged to phylogenetic group B2 and serotypes O75:H5 and ST1193. The strains in this cluster were associated with UTIs, uEC strains, and complicated UTIs (cUTI) and were mainly localized in the bladder (Figure 3). The correlation of the following frequencies: disease vs. STs, disease vs. region, and disease vs. number of plasmids, are shown through heat maps (Supplementary Figure S6).

#### 3.2.3 Analysis of the UPEC strain STs

**Figure 4:**
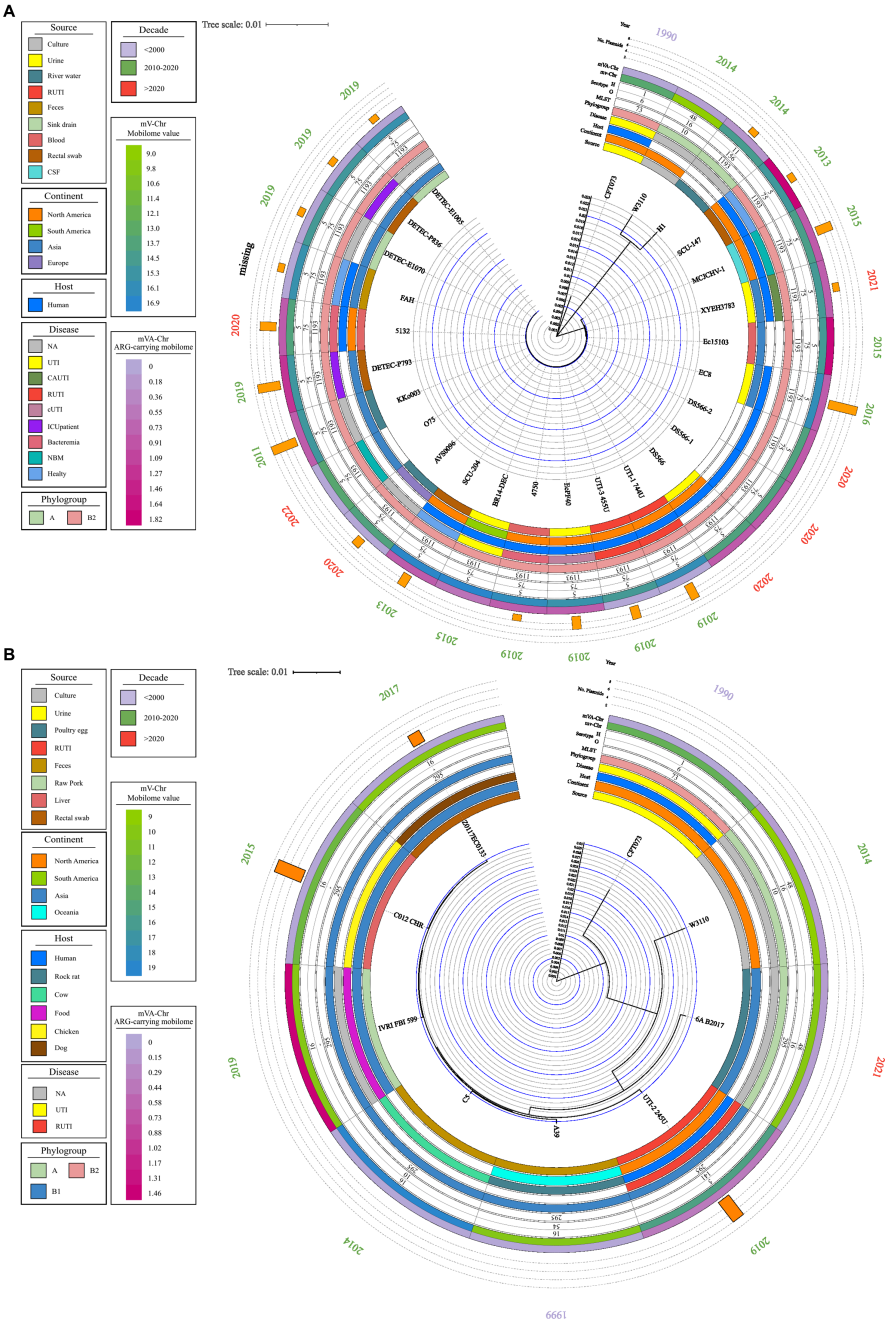
Phylogenetic map of ST1193 and ST295. A phylogenetic map was prepared using the complete genome using MAUVE and Geneious Prime. Data on source, disease, and continent of origin are presented, following the color code in the legend key. The year of isolation, phylogroup, serotype, ST, and the number of plasmids in each genome are also shown. **(A)** Phylogenetic tree of ST1193. **(B)** Phylogenetic tree of ST295.

From the comparative genome analysis, the UTI-1_774 U and UTI-3_455 U strains strongly correlated with 24 *E. coli* strains from different origins. These strains belonged to ST1193, a widely-distributed clone that is a causative agent of human infections and healthcare-associated infections (HCAIs) in hospitals around the world. The ST1193 clone belongs to the B2 phylogenetic group and the O75:H5 serotype. Generally, this clone can be isolated from RUTIs cultures, cerebrospinal fluid (CSF), blood, catheter cultures, and it is also present in healthy individuals. The bioinformatic analysis showed a high proportion of genes associated with the mobilome in strain ST1193; however, the proportion of genes associated with resistance was low (Figure 4A).

The UTI-2245 U strain showed a strong association with six *E. coli* strains of different origins belonging to ST295 (Figure 2B). Comparative genome analysis indicated that this clone has an animal origin, mainly domestic and farm animals. The ST295 strains belong to two commensal phylogenetic groups (B1 and A), three serogroups (O10, O16, and O141), and the flagellar variant H16. Strains belonging to ST295 contained a low proportion of genes associated with the mobilome; in contrast, a high proportion of genes was associated with resistance in strains belonging to ST1193 (Figure 4B).

#### 3.2.4 Comparison of complete UPEC strain genomes

**Figure 5:**
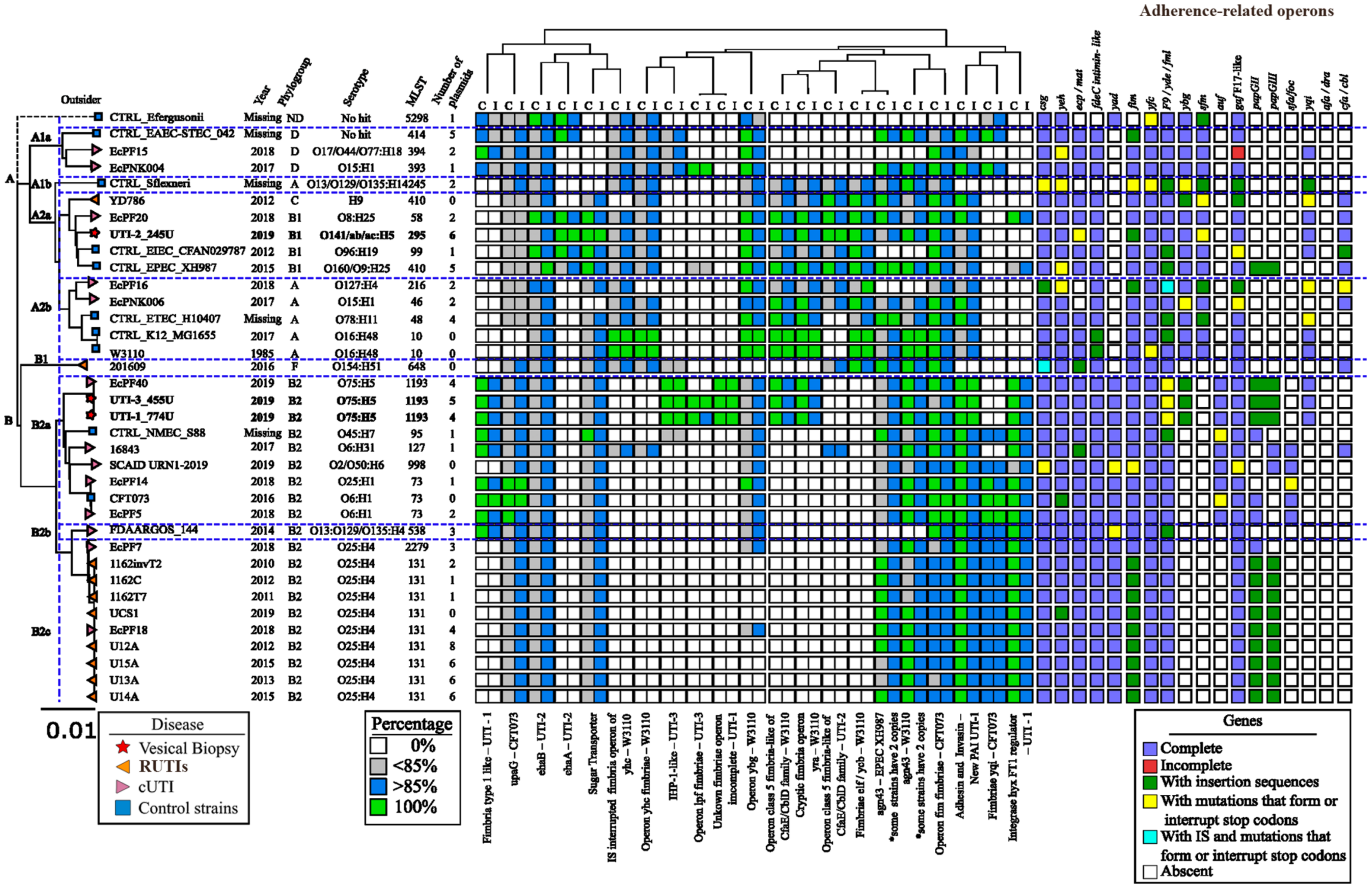
Identification of genes and operons associated with adherence in *E. coli* strains. The values obtained by BLAST of the regions of interest are presented. The percentage of identity or coverage is represented in colored boxes using the color code shown in the “Percentage” legend key. In the analysis of the operons associated with fimbriae, each box represents an operon, and the color following the key present in the legend “Operons” shows if it is complete, partial, or contains point mutations that generate premature stop codons or insertion sequences.

The phylogenetic tree was built from the three clinical strains of RUTIs of bladder biopsy culture included in this study, 13 cUTI genomes, 10 RUTI genomes, and 11 control genomes, all previously described in this study. The phylogenetic tree of the 36 genomes generated with MAUVE and Geneious identified *E. fergusonii* as a clonally unrelated strain (outlier). Moreover, 97.22% (35/36) of the remaining genomes showed 99.99 identity, and two main clades were generated. Clade A included the control strains of intestinal origin (EAEC_STEC_042, EIEC_ CFAN029787, EPEC_ XH987 and ETEC_ H10407), *S. flexneri* and two *E. coli* K12 strains (K12_MG1655 and *E. coli* W3110) (Figure 5). The genomes in clade A shared the following regions: operon *yhE* fimbria-W3110, operon *ybg* -W3110, operon class 5 fimbria-like of *cfaE*/*cblD* family-W3110, operon cryptic of the fimbria *yra*-W3110, operon class 5 fimbria-like of *cfaE*/*cblD* of UTI-2, fimbria *elf*/*ycvb* -W3110, operon *fim* fimbriae of CFT073, and adhesin and invasin-New PAI of UTI-1. Interestingly, the *csg*, *yeh*, *ecp/mat*, *fdeC intimin-like*, *fim*, *yfc*, *F9/yde/fml*, *ybg*, *sfm*, *gaf-F17-like*, *yqi*, *and cfa/cdI* operons were grouped together in the clade; however, in this clade, the *yad*, *auf*, *pap*, *sfa/foc*, and *afa/drA* operons were not identified. Clade A contains genomes mainly associated with phylogroups A and B1. The genomes associated with this clade showed a heterogeneous distribution of serotypes and STs (Figure 5).

Clade A was divided into four subclades, A1, A2a, A2b and A2c. Clade A1 was made up of three strains belonging to phylogenetic group D, and the intestinal strain EAEC_STEC_042 was included. Clade A2a contained *Shigella flexneri*. Clade A2b included the RUTIs UTI-2_245U and YD786, a cUTI EcPF20, EIEC_CFAN029787, and EPEC_XH987. The genomes of this clade mainly belonged to phylogenetic group B1 and were associated with the *ehaA* adhesin and the *cfa*/*cbi* fimbriae operon. In this clade, the RUTI strain UTI-2_245U had a high number of plasmids. Two cUTI genomes (Ec PF16 and EcPNK006) and three control strains (ETEC_ H10407, K12_MG1655, and *E. coli* W3110) were grouped into A2c. This clade did not have homologous regions of interest, and the genomes were characterized by the presence of intimin-like *fdeC,* which were absent in the control genomes. However, fimbrial operons were absent, denoted by *ecp*/*mat*, *gaf* F17-like (Figure 5).

Clade B was mainly characterized by the presence of nonhomologous regions of interest. In these regions, genes encoding the type 1 fimbria-lke of UTI-1, Adhesin-Invasin New PAI of UTI-1, *yqi* fimbria of CFT073, and regulatory integrase of type 1 fimbria *hxy* of UTI-1 were found. Clade B was made up of 21 urinary *E. coli* strains (8 cUTI strains, 11 RUTIs strains, one NMEC_S88 and UPEC CFT073), 95.23% (20/21) belonging to phylogenetic group B2 and 71.42% (15/21) showing three ST types [14.28% (3/21) for ST73, 42.85% (9/21) for ST131, and 14.28 (3/21) for ST1193]. Interestingly, the genome of RUTI 201609 categorized into phylogenomic group F was not included in clade B with the other RUTIs, genomes (Figure 5).

Clade B is divided into three subclades, B1, B2a, and B2b. Clade B1, containing only strain 201,609, is characterized by the presence of the *cfa*/*cbi* operons; however, there is an absence of the *ybg* and sfm fimbrial operons. Clade B2a includes the strains UTI-1_774U and UTI-3_455U, characterized by the element’s fimbria type 1-like of UTI-1, *upaG* of CFT073, *ehaB* of UTI-2, sugar transporter, operon *ygb* of W3110, Agn43 of EPEC_XH987, Adhesin-Invasin New PAI of UTI-1, and the regulatory integrase of the type 1 fimbria *hxy* of UTI-1. Additionally, the genomes of this clade had the fimbrial operons *csg*, *yeh*, *ecp/mat*, *fdeC intimin-like*, *yad, fim*, *yfc*, *F9/yde/fml*, *auf,* and *gaf-F17- like*. The strains in clade B2a were mainly from serotype O75:H5/ST1193 [33.33% (3/9)]. The B2b clade mainly included the recurrent strains and is characterized by having the same pattern of nonhomologous regions of interest and fimbrial patterns strains, but does not have the fimbria type 1-like of UTI-1, operon *ygb* of W3110 and *papG*II and *papG*III fimbrial operons. The strains in clade B2b were mainly from serotype O25:H4/ST131 [81.81% (9/11)] (Figure 5).

#### 3.2.5 Phenotypic analysis of the antibiotic resistome in the three RUTI-associated UPEC strains

The susceptibility profile to 14 categories of antibiotics allowed us to determine the phenotypic antibiotic resistome in the UPEC strains associated with RUTIs. The UPEC strain UTI-1_774U displayed an MDR-6 profile (multidrug resistance to 6 antibiotic categories including penicillin, β-lactam combination agents, lipopeptides, macrolides, quinolones and fluoroquinolones, and folate pathway antagonists). The UPEC strain UTI-2_245U showed an MDR-5 profile (multidrug resistance to 5 antibiotic categories including β-lactam combination agents, lipopeptides, macrolides, tetracyclines, and folate pathway antagonists). The UPEC strain UTI-3_455U showed an MDR-3 profile (multidrug resistance to 3 antibiotic categories including lipopeptides, macrolides, quinolones, and fluoroquinolones). None of the three UPEC strains were phenotypic ESBL producers (Table 3).

**Table 3:** Phenotypic and genotypic analysis of the antibiotic resistance profile of recurrent UPEC clinical strains.

Strain	UTI-1_774U	UTI-2_245U			UTI-3_455U	
No. of elements resistance associated	84	86			80	
Efflux pumps genes	50	53			51	
Unique elements	*aph(6)-Id, aph(3'')-Ib, dfrA17, tem-1*	*sat-1, mdtM, tetD, CMY-60, tetA, ant(3'')-Ia, dfrA1, tetR*			None	
ESBL	Non–ESBL-producing	Non–ESBL-producing			Non–ESBL-producing	
Antimicrobial categories	Sc	Genes	Sc	Genes	Sc	Genes
Penicillin’s	R	*ompF*	S	*ompF*	S	*ompF*
β-lactam combination agents	R	dfrA17*	R	dfrA1*	S	
Cephems		tem-1*		*CMY-60,*		
cephalosporins II	S		S		S	
cephalosporins III	S		S		S	
cephalosporins IV	S		S		S	
Monobactams	S		S		S	
Carbapenems	S		S		S	
Lipopeptides	I	*arnA, bacA, mgrB, phoP, pmrBCEF*	I	*arnA, bacA, mgrB, phoP, pmrBCF*	I	*arnA, acA, mgrB, hoP, pmrBCEF*
Aminoglycosides	S	APH(3'')-Ib*, APH(6)-I*, fyuA, kdpE	S	kdpE, ANT(3'')-Ia, ANT(3'')-Ia*	S	*KdpE, fyuA*
Macrolides	R	mphA*, mrx	R		R	mphA*, mrx
Tetracyclines	S	acrR*	R	*tetADR*	S	acrR*
Glycylcyclines	S		S		S	
Quinones and fluoroquinolones	R	*gyrA, mfd, parCE*	S	*gyrA, mfd, parCE*	R	*parCE, gyrA, mfd*
Folate pathway antagonist	R	folP, leuO, sul2*	R	folP, leuO, sul2*	S	*folP, leuO*
Phenicols	S		S		S	
Fosfomycin	Ne	*glpT, murA, uhpT*	Ne	*glpT, murA, uhpT*	Ne	*glpT, murA, uhpT*
Nitrofurans	S	*nfsA*	S	*nfsA*	S	*nfsA0*
Profile	MDR-6	MDR-5			MDR-3	

No: number; Sc: susceptibility; R: resistant; S: sensitive; I: intermediate; Ne: non-evaluated; MDR: multidrug resistant; *: plasmidic gene. Penicillins (ampicillin); β-lactam combination agents (amoxicillin/clavulanate and piperacillin-tazobactam); Cephems [Cephalosporins 2 (cefaclor and cefoxitin), cephalosporins 3 (ceftazidime, cefotaxime, and ceftriaxone), cephalosporins 4 (cefepime)]; Monobactams (aztreonam); Carbapenems (imipenem, doripenem, ertapenem, and meropenem); Lipopeptides (colistin); Aminoglycosides (gentamicin and amikacin); Macrolides (erythromycin); Tetracyclines (tetracycline); Glycylcyclines (tigecycline); Quinolones and flouroquinolones (ciprofloxacin and ofloxacin); Folate pathway antagonist (trimethoprim-sulfamethoxazole); Phenicols (chloramphenicol); Nitrofurans (nitrofurantoin).

*ANT* (aminoglycoside nucleotidyltransferase); *APH* (aminoglycoside phosphotransferase); *arnA* (UDP-4-amino-4-deoxy-L-arabinose formyltransferase); *bac* (undecaprenyl-diphosphatase); *CMY* (class C beta-lactamase cephamycins resistance-related); *dfr* (dihydrofolate reductase); *fol* (dihydropteroate synthase); *fyu* (ferric yersiniabactin uptake); *glp* (glycerol-3-phosphate); *gyr* (gyrase); *kdp* (potassium uptake regulator); *leu* (leucine response transcription factor); *mfd* (mutation frequency decline transcription-repair coupling factor); *mgr* (magnessium responsive promoter); *mph* (macrolide phosphotransferase); *mrx* (macrolide inactivation gene with unknow function); *mur* (UDP-N-acetylglucosamine 1-carboxyvinyltransferase); *nfs* (oxygen-insensitive NADPH nitroreductase); *omp* (outer membrane porin); *par* (DNA topoisomerase); *pho/pmr* (phosphoethanolamine transferase); *sul2* (sulfonamide resistant dihydropteroate synthase); *TEM,* (“Temoniera” Class A beta-lactamase); *uhp,* (hexose phosphate transport).

The resistome analysis of the strains UTI-1_774U, UTI-2_245U, and UTI-3_455U presented 84, 86, and 80 genetic elements associated with antibiotic resistance, respectively. Additionally, 59.52% (50/84) for UTI-1, 61.62% (53/86) for UTI-2, and 63.75% (51/80) for UTI-3 display mobile elements associated with active efflux pumps. In the UTI-1_774U strain, unique genetic elements were found, such as aminoglycoside O-phosphotransferase (*APH(6)-Id*), aminoglycoside 3′-phosphotransferase (*APH(3″)-Ib*), integron-encoded dihydrofolate reductase of *E. coli* (*dfrA17*), and *TEM-1*. In the UTI-2_245U strain, the following unique genetic elements were found: *sat1*, *mdtM*, *tetD*, *CMY-60*, *tetA*, *ANT (3″)-1a*, *dfrA1*, and *tetR*. No unique genetic elements were identified in the UTI-3_455U strain (Table 3).

The three UPEC strains were characterized by a resistome with genes for outer membrane proteins (*ompF*) associated with resistance to penicillin (*arnA*, *bacA*, *mgrB*, *phoP*, and *pmrBCF*) and lipopeptides. The genes for aminoglycoside phosphotransferase enzymes [*APH (3″)-1b*, *APH(3″)-Ib*, *ANT (3″)-1a*], and *kdpE* were associated with resistance to aminoglycosides. Moreover, the *gyrA*, *mfd*, and *parCE* genes conferred resistance to quinolones and fluoroquinolones in the UPEC strains. Resistance to fosfomycin was related to the presence of the *glpT*, *murA*, and *uhpT* genes. Likewise, the *nfsA* gene was found to be associated with resistance to nitrofurans (Table 3).

The genome analysis of the UTI-1_774U strain showed that the *TEM-1* gene encodes an ESBL, and the genome analysis of the UPEC strain UTI-2_245U showed that the *CMY-60* gene encodes a β-lactamase resistant to cephamycin. Macrolide resistance was associated with the *mphA* and *mrx* genes in the UTI-1_774U and UTI-3_455U strains. However, in the genome of the UTI-2_245U strain, genes related to its resistance to macrolides were not identified. Resistance to tetracycline antibiotics was mainly associated with the *acrR* gene in the UTI-1_774U and UTI-3_455U strains and with the *tetADF* operon in the UTI-2_455U strain. The *folP* and *leuO* genes, which confer resistance to folate pathway antagonists, were identified in the genomes of the three UPEC strains. However, only strains UTI-1_774U and UTI-3_455U presented integron-encoded dihydrofolate reductase (*dfrA17* and *dfrA1*) and *sul2* genes for resistance to folate pathway antagonists and sulfonamides. The three strains, UTI-1_774U, UTI-3_455U, and UTI-2_455U, were phenotypically and genotypically sensitive to cephems and cephalosporins, monobactams, carbapenems, glycylcyclines, and phenicols (Table 3). The Spearman correlation coefficient showed a non-relationship between the virulence genes and the resistance genes of the 172 *E. coli* sequences (Supplementary Figure S7).

#### 3.2.6 Genetic elements associated with UPEC resistance

**Figure 6:**
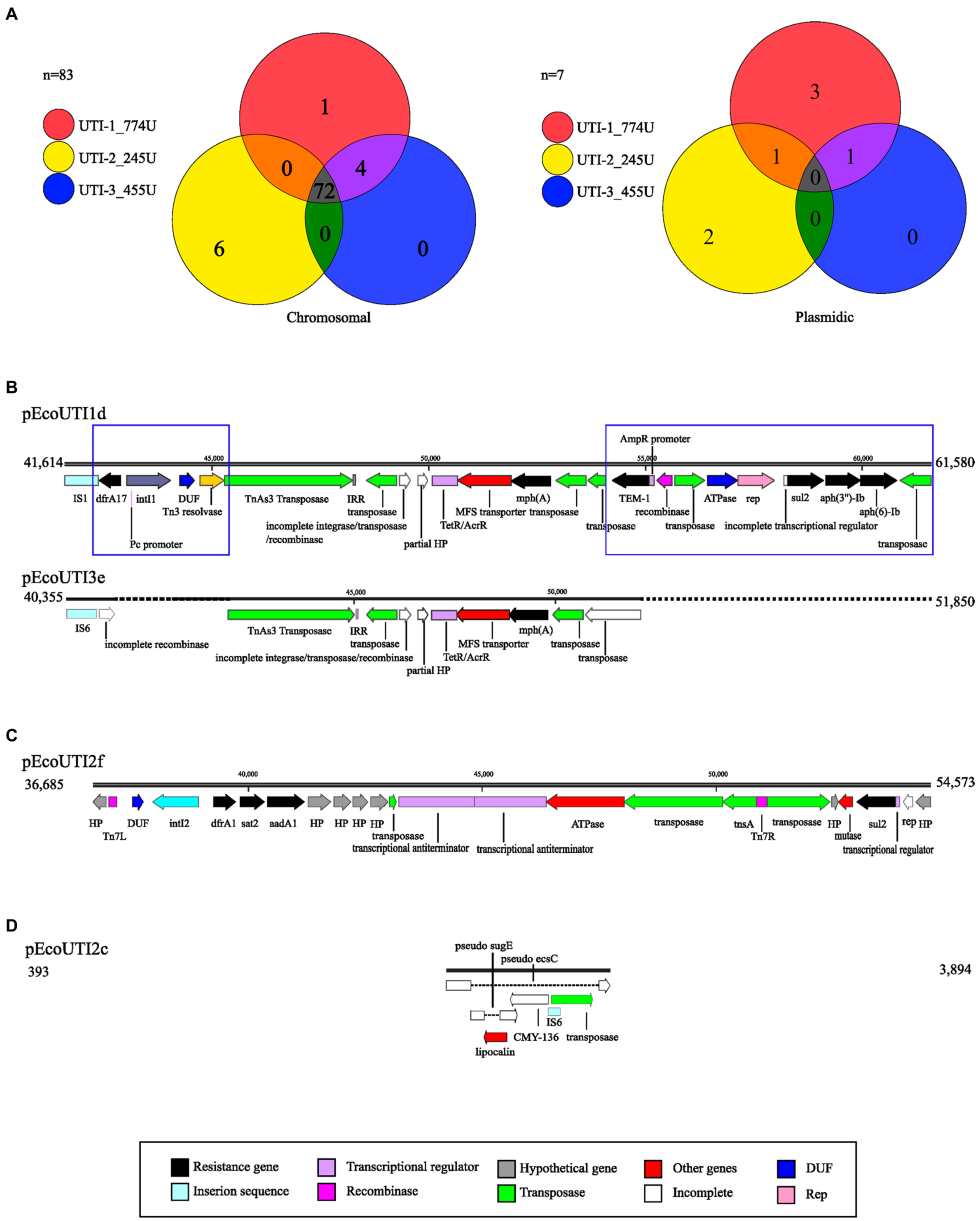
Genetic elements associated with resistance (GEAR). **(A)** Venn diagrams representing common and independent GEAR found with CARD on the chromosome and plasmids of UPEC strains. Each strain is represented with a circle of the corresponding color. **(B)** Alignment comparing the resistance-carrying region of plasmids pEcoUTI1d and pEcoUTI3e. **(C)** Representation of the resistance-carrying region of the pEcoUTI2f plasmid. **(D)** Representation of the resistance carrier region of the pEcoUTI2c plasmid. The coding sequences are shown in colors following the legend key.

A comparison of the results using the CARD database showed 72 genetic elements associated with antibiotic resistance (GEAR) that were shared by the three UPEC strains included in this study (Table 3). The *sul2* gene was shared between strains UTI-1_774U and UTI-2_245U, while 5 GEARs (*mrx*, *pmrE*, *fyuA*, *mphA*, and *vgaC*) were identified in the two strains UTI-1_774U and UTI-3_455U. In addition, eight unique GEARs (*CMY-60*, *aadA*, *dfrA1*, *mdtM*, *sat1*, *tetA*, *tetD*, and *tetR*) were identified in strain UTI-2_245U, and four unique GEARs (*APH(3″)-Ib*, *APH(6)-Id*, *TEM-1*, and *dfrA17*) were identified in strain UTI-1_774U (Table 3; Figure 6A).

Additionally, a comparative analysis of the strains that displayed a GEAR (*sul2*) within their genomes revealed that they shared two plasmids, pEcoUTI1d from UTI-1_774U and the plasmid pEcoUTI2f from UTI-2_245U. A GEAR (*mphA*) was also identified between the plasmids pEcoUTI1d and pEcoUTI3e from the strains UTI-1_774U and UTI-3_455U, respectively. Four unique GEARs (*dfrA17*, *TEM-1*, *aph(3″)-Ib*, and *aph(6)-Id* in pEcoUTI1d) for UTI-1774 U and 2 GEARs (*dfrA1*, *sat2*, and *aadA1* in pEcoUTI2f) in the UTI-2_245U strain were identified (Figure 6A).

#### 3.2.7 Antibiotic resistance plasmids in UPEC strains

With SnapGene software, four resistance-carrying plasmids were identified (pEcoUTI1d, pEco UTI3e, pEcoUTI2f, and pEcoUTI2c; Figures 6B–D). The analysis of the alignment of the region between 41,614 bp and 61,580 bp in the plasmid pEcoUTI1d of the strain UTI-1_774U and the region from 40,355 bp to 51,850 bp in the plasmid pEcoUTI3e of UTI-3_455U showed 86.2% similarity between them. These two regions were identified within a mobile element of ~40,000 bp flanked by the *lolE* and *mer* genes. The differences between these regions showed two insertions: (1) An insertion of a 3,347 bp fragment located between an IS1 insertion sequence and a TnAs3 transposase. The resistance gene *dfrA17*, a class 1 integrase, and a Tn3 resolvase were also identified in this insertion. (2) A 9,489 bp insertion fragment harboring the resistance genes *TEM-1*, *sul2*, *aph(3″)-Ib*, and *aph(6)-Ib* and the rep replication site is located between two transposases (Figure 6B).

A 17,888 bp region between the *lolE* and *mer* genes (36,865 bp to 54,573 bp) is in the plasmid pEcoUTI2f of the UTI-2_245U strain. This region was characterized by harboring the resistance genes *dfrA1, sat2, aadA1, sul2*, and a class I integrase. Several genes that encode hypothetical proteins, transposons, transcriptional regulators, and a partial fragment of the rep replication region were identified (Figure 6C). Finally, the plasmid pEcoUTI2c in the strain UTI-2_245U showed a region of 3,501 bp (393 bp to 3,894 bp), with a nonfunctional gene for the β-lactamase *CMY-136*, which is inactivated by insertion sequences. In this region, the *sugE* and *ecsC* pseudogenes were also inactivated by insertions (Figure 6D).

#### 3.2.8 Genotypic and phenotypic analysis of the flagellum in clinical strains of UPEC

After incubation in mobility medium at 37°C in a 5% CO_2_ incubator for 18 h, the UTI-2_245U strain showed a mobility halo of 65 mm in diameter, while the positive control strain UPEC CFT073 exhibited a halo of 32 mm in diameter. However, the strains UTI-1_774U and UTI-3_455U in addition to the negative control *E. coli* W3110 strain did not show motility halos (Figure 2C). The development of flagellar mobility as a function of time showed that the UTI-2_245U and CFT073 strains began to develop mobility halos after 3 h of incubation. Moreover, the qualitative mobility analysis showed a more pronounced mobility curve and a higher speed than the UPEC control strain CFT073 (Figure 2D). The TEM micrographs of the UTI-2_245U and CFT073 strains cultured in pleuropneumonia-like organism (PPLO) medium showed long, thick, flexible, and polar structures suggestive of the presence of flagella. However, TEM micrographs of the UTI-1_774U, UTI-3_455U, and *E. coli* W3110 strains grown under the same conditions did not show these characteristic flagella structures (Figure 2E). Although bioinformatic analyses were mainly used in this study, the genomes showed the presence of genes associated with flagellar biogenesis, which are shown in Supplementary Table S1; Supplementary Figure S8.

## 4 Discussion

UTIs are prevalent worldwide and frequently cause outpatient and emergency medical interventions. Treating and controlling UTIs is a costly challenge for public health systems around the world, and they are one of the most common reasons for the use of broad-spectrum antibiotics (Sihra et al., 2018). The average recurrence rate of UTIs in women has been reported to be more than 30–40% (Kwok et al., 2022). The duration of an RUTI can be variable; however, in most women, recurrences occur between 6 and 12 months (Jung and Brubaker, 2019). The three patients in this study experienced several recurrence periods per year over a period of 1–3 years before undergoing a bladder biopsy. Adult women with RUTIs may present with very variable clinical symptoms, which reduce their quality of life and require individualized attention. RUTIs can occur due to an unresolved infection, reinfection, or relapse, and it is estimated that approximately 80% of RUTI cases in women occur as a result of reinfection (Pigrau-Serrallach, 2005). The clinical history recovered from the patients indicated chronic urinary infection (Chamoun et al., 2020), follicular cystitis (Mateos Blanco et al., 2007), and interstitial cystitis (Warren et al., 2008), clinical entities that may be related to RUTI episodes. The risks factors for an RUTI are essential since they said the selection of a supportive nonantibiotic treatment. Risk factors frequently implicated in the development of an RUTI have included diabetes, recent sexual intercourse, history of urogenital surgery, urinary emptying dysfunction, accidental intestinal leakage, and urinary incontinence (Sihra et al., 2018; Jung and Brubaker, 2019; Ahmed et al., 2023), as observed in the clinical data of the patients in this study. In older adult women, these comorbidities can be complicated by pyelonephritis (1.6%) or urosepsis (0.08%), conditions that put the patient’s life at serious risk (Bradley et al., 2022).

In Mexico, the high incidence of obesity (42.2%) and diabetes mellitus (29%) is a political, social, and public health phenomenon that directly affects the prevalence and prognosis of RUTIs and requires new treatment strategies. Jointly, the patients in this study were subjected to topical nonantibiotic and hormone replacement treatments as an alternative to reduce symptoms and possibly eradicate the infection. These three patients received treatments such as intravesical installation of hyaluronate, oral lactobacilli, anticholinergics, topical oestrogens, and behavioral therapy. However, the alternative treatments were only effective during the application time. Various nonantibiotic management options for RUTIs have been frequently reported, with variable results depending on the progression of the RUTI, the patient’s age, and the use of previous treatment (Sihra et al., 2018).

Microbiological analysis of urine cultures has been considered the “gold standard” in the diagnosis of an RUTI; however, its diagnostic accuracy has been very poor (Kwok et al., 2022). Microbiological procedures have allowed the recovery of clinical UPEC strains obtained from biopsy tissue during an RUTI episode. Bladder biopsy tissue obtained by transurethral cystoscopy has rarely been used to diagnose UTIs (Sycamore et al., 2014). However, although this invasive process has not been wholly accepted to diagnose UTIs, it has been used to diagnose interstitial cystitis (Trifillis et al., 1995; Natale et al., 2022). In this study, biopsies of bladder epithelium and urine samples were obtained from 3 patients diagnosed with RUTI for culture and isolation of UPEC strains. The presumptive evaluation of the similarity between urine strains and biopsies from the same patients was carried out by PFGE for epidemiological surveillance and the complete genome sequencing for determining the clonal relationship between strains (Blankenship et al., 2023). In this work, PFGE allowed us to observe clonality between urine and biopsy strains from the same patient. Moreover, our work is the first WGS study on UPEC strains obtained by bladder biopsy tissue from adult women with a history of RUTI for several years. The UPEC strains were analyzed with WGS molecular technology to improve diagnosis and inform the decision-making process for the treatment of patients with RUTIs. WGS has allowed the comparison of UPEC bacterial genomes from patients with complicated UTIs, uncomplicated UTIs, and RUTIs. However, even with complete genome data, it has been difficult to identify genetic characteristics associated with a specific clinical condition. This concern has been previously reported, and international organizations have made efforts to harmonize the genomics databases (Anagnostopoulos et al., 2022). WGS has facilitated the identification of common clones, clonal complexes, and essential serotypes between animals and humans, suggesting a bidirectional clonal transfer as an essential transmission method of bacteria (Flament-Simon et al., 2020). In closed environments, such as retirement homes for elderly individuals, WGS has identified RUTIs caused by the same strain of UPEC (Hidad et al., 2022). In our study, the three genomes of the UPEC strains were very closely related, observing an identity of 99% between UTI-1_774U and UTI-3_455U and an identity of 88–93% when compared with the strain UTI-2_245U. The genomic analysis of the three strains associated with RUTIs showed areas of low similarity, with loss of identity and coverage in the genes encoding pathogenicity islands (PAIs), compared to the genes encoding PAIs in the UPEC control strain CFT073. The characterization analysis of the *E. coli* strains suggests that the capacity to colonize new niches during intestinal and extraintestinal pathologies is closely associated with the presence/absence of widely recognized colonization, virulence, and fitness factors (Kaper et al., 2004). These factors are frequently grouped into PAIs and are responsible for causing a specific clinical condition in a susceptible host (Desvaux et al., 2020). Our findings suggest that RUTI strains did not present a specific genomic characteristic differentiating them from other pathotypes. We hypothesize that the loss of VFs results in the attenuation of the strains and favors the development of a chronic infection in the lower urinary tract. Furthermore, the acquisition of genes by horizontal transfer was a common element among the RUTI strains in our study, showing numerous plasmids, insertion sequences, prophages, and PAIs. The UTI-2_245U strain showed multiple CRISPR arrays, which were not related to virulence or resistance factors; however, they represented elements with inherent potential to be platforms that contribute to genomic plasticity and the horizontal acquisition of genetic material, which may have clinical relevance among different isolates (Rozwadowski and Gawel, 2022).

The comparative genomics together with the creation and curation of a database of 158 complete sequenced genomes of human urinary *E. coli* (uEc) strains allowed us to analyze our RUTI strains in evolutionary, geographical, and pathogenic contexts. The genomic analysis showed that the uEc strains are closely related (99.95% similarity), grouping into clades of intestinal and extraintestinal pathotypes; likewise, they were grouped into subclades based on their STs and serotypes (Valiatti et al., 2020; Schüroff et al., 2021). However, no relationship with the clinical conditions to which they were related was observed (Matamoros et al., 2017; Flament-Simon et al., 2020). The strains of uEc, cUTI, and RUTI in other studies were mainly associated with the O25:H4/ST131 clones, producers of ESBL and MDR that are widely distributed and cause extraintestinal infections (Contreras-Alvarado et al., 2021). The strains UTI-1_774U and UTI-3_455U were independently clustered, with the closest control genome being that of neonatal meningitis-associated *E. coli* S88 (Peigne et al., 2009), while the strain UTI-2_245U clustered together with the control genome of enterohaemorrhagic *E. coli* Sakai (Makino et al., 1999). The strains UTI-1_774U and UTI-3_455U belonged to phylogenetic group B2, serotype O75:H5/ST1193, and were resistant to fluoroquinolones. This clone has been reported in Spain, Europe, North America, and various parts of the world (García-Meniño et al., 2022; Pitout et al., 2022) as a pathogenic and MDR emerging clone in humans (probability ≥92%). This clone has been associated with community UTIs and bloodstream infections (García-Meniño et al., 2022; Pitout et al., 2022). The UPEC strains UTI-1_774U and UTI-3_455U of Mexican origin presented a set of chromosomal mutations (*gyrA* S83L, *gyrA* D87N, *parC* S80I, and *parE* L416F) that conferred resistance to fluoroquinolones. These strains presented many genomic characteristics related to RUTIs and not community UTIs similar to those reported by García-Meniño et al. (2022). The results suggest that clonal lineages related to uncomplicated and community-based UTIs may have pathogenic characteristics to adapt to recurrent urinary strains if the appropriate environmental and host factors are present. Interestingly, UTI-1_774U and UTI-3_455U were related to the clinical cUTI strain named EcPF40 in the database, reported by Sharon et al. (2020) to be associated with RUTI. Our work supports the proposal to monitor ST1193 as a new clone at the same level of importance as ST131 (Pitout et al., 2022). The UTI-2_245U strain was grouped into the phylogroup B1 and serotype O141:H5/ST295. This clone of mainly Asian origin was grouped with the controls of intestinal origin and allowed us to infer that this clade is transitional between strains from intestinal infections and extraintestinal strains (Fuentes-Castillo et al., 2023). Previously, it has been reported that slight changes in the core genome can result in changes in a strain from an intestinal environment to a highly specialized extraintestinal pathotype (McNally et al., 2013). ST295 is associated with animal hosts, giving this ST a strong zoonotic component. Fuentes-Castillo et al. (2023) reported the association of ST295 clones with seagulls; the authors conclude that the migratory routes of these animals can contribute to the dissemination of these clones in new environments (Fuentes-Castillo et al., 2023). The clones of ST295 have been identified in hospital wastewater samples, suggesting that UTI-associated bacteria play a role in emerging antibiotic-resistance genotypes in hospital settings. A high diversity of STs and phylogenetic groups with pathogenic lineages can increase hospital and community dissemination (Davidova-Gerzova et al., 2023). The discovery of clone ST295 in a patient with RUTI supports the urgent need to monitor the distribution of this clone and its correlation with other possible reservoirs.

Manual analysis of virulence and fitness factors allowed the identification of various partial, incomplete, inactive, or absent operons and regions of interest associated with adherence. The role of fimbriae as the main factors in adherence, colonization, and invasion of *E. coli* to the urothelium was of great importance in this work (Desvaux et al., 2020; Hozzari et al., 2020). The comparative genomic analysis of the fimbrial operons in 10 RUTIs genomes, 13 cUTI genomes, and 10 control genomes of intestinal and extraintestinal *E. coli* strains showed the grouping of the strains UTI-1_774U and UTI-3_455U in the clade shared by extraintestinal strains and the UTI-2_245U strain with the intestinal strains. Interestingly, the analysis revealed the absence of the *pap* operon, located in PAI I_CFT073_, in 88.5% of the strains. P fimbriae are vital for adherence to the renal epithelium and is associated with invasive *E. coli* strains (Biggel et al., 2020). However, the analysis showed that the RUTIs strains only conserved the regulatory gene *papX*, which has participated as an inhibitor of flagellar mobility (Simms and Mobley, 2008) and whose inhibitory effect may be related to the negative result in the mobility assays with the UTI-1_774U and UTI-3_455U strains. The genomic analysis of the UTI-2_245U strain showed a loss of genes associated with type 1 fimbriae (FT1). FT1 is the most relevant classic factor in the invasion of UPEC to the urinary tract (Eto et al., 2007). Nevertheless, ablation of FT1 did not wholly abate invasion in the *in vitro* assays, and it has been observed that the ECP (*E. coli* Common Pilus) fimbriae can promote invasion in the mouse bladder (Saldaña et al., 2014). In this context, the presence of other fimbriae operons similar to FT1, such as the F9 fimbriae, which is upregulated 6-fold in cUTI (Conover et al., 2016) and F17-like, which promotes the formation of reservoirs in the intestine (Spaulding et al., 2017), may contribute to reinfections and favor the establishment of UPEC strains. Data with the UPEC reference strain CFT073 revealed the presence of 10 types of fimbriae belonging to the chaperone-accommodator family and two related to type IV pili (Welch et al., 2002). Previous studies have confirmed the role of fimbria: fimbria type 1 (*fim*) (Martinez, 2000), fimbria P (*pap*) (Kuehn et al., 1992), fimbria F1C/S (*foc/sfa*) (Khan et al., 2000), and AFA/DRA fimbria (*afa/dra*) (Selvarangan et al., 2004) in the pathogenicity of UPEC to the urinary tract. The findings in this work indicate that fimbriae 1, P, F1C/S, and AFA/DRA are rare in cUTI and RUTI strains. Miyazaki et al. (2002) reported that fimbriae 1, P, and S are not essential for UPEC colonization in the urinary tract. The results obtained in this work suggest that the high diversity of fimbrial operons and even the participation of other surface proteins, such as FimA, BtuB, ChuA, FepA, FyuA, UidC, NmpC, OmpA, OmpC, OmpF, OmpT, Flu, CarB, and MdhK are expressed during the growth of *E. coli* in urine, and in the colonization of the urinary tract (Wurpel et al., 2016). Other fimbrial operons identified with high frequency in the genomes of cUTI, RUTIs, UTI-1_774U, UTI-2_245U, and UTI-3_455U were the Curli operon (*csg*) (Luna-Pineda et al., 2019) and Ecp operon (Stacy et al., 2014), Yfc operon (Spurbeck et al., 2012), Yeh operon (Ravan and Amandadi, 2015), and F17-like operon (Martin et al., 1997), which are considered nonclassical virulence factors of urinary strains. Toxins are virulence factors associated with colonization of the urinary tract. In addition, they allow the dispersion of bacteria to tissues, promote the lysis of host cells, facilitate the release of micronutrients such as iron, which will subsequently be transported by siderophores (Dhakal and Mulvey, 2012), and increase neutrophil survival (Los et al., 2013). The *clbA* and *clbQ* genes that encode a colibactin, the *pic* gene that encodes a serine protease, the *vat* gene that encodes a hemoglobin protease, and the *hlyA* gene for α-haemolysin are factors widely described in the UPEC reference strain CFT073 (Luo et al., 2009).

The RUTI strains UTI-1_774U and UTI-3_455U did not present the exotoxins Hly, Cnf, Ast, Cdt, and Clb or the toxins Sat, Vat, and Tsh. Moreover, the UTI-2_245U strain did not present any genes associated with these toxins. As a result, we can suggest that the absence of classic UPEC VFs seems to promote the chronicity of these strains. The results allow us to hypothesize that toxins are not needed by invasive UPEC strains, mainly because they can have free access to iron in the host cell cytoplasm. Attenuation of toxicity appears to contribute to the chronic state of the infection. Similar behavior has been observed in the adaptive process of *Staphylococcus aureus* in its colonization in the nose, where various mutations promote the attenuation of cytotoxic activity and the generation of a nonhemolytic phenotype, which allows infection with reduced clinical manifestations, promoting a severe disease (Das et al., 2016). The strains UTI-1_774U, UTI-2_245U, and UTI-3_455U were shown to be resistant to penicillin, folate pathway antagonists, quinolones, and fluoroquinolones, with no resistance to cephalosporins. Furthermore, strain UTI-1_774U was MDR-6, UTI-2_245U MDR-5, and UTI-3_455U MDR-3. UPEC strains associated with RUTI have been previously reported (Luo et al., 2012; Al-Mayahie and Al Kuriashy, 2016; Issakhanian and Behzadi, 2019; Karami et al., 2021). In this study, MDR in the strains UTI-1_774U, UTI-2_245U, and UTI-3_455U was related to mobile genetic elements (MGEs). The ESBL gene, *TEM-1*, confers resistance to penicillin and cephalosporins; the genes *APH(3″)-Ib*, *APH(6)-I*, and *APH(3″)-Ia*, which confer resistance to aminoglycosides; the hydrofolate reductase genes *dfrA1* and *dfrA17*, which confer resistance to trimethoprim; the *sul2* gene, which confers sulfonamide resistance; and *the mphA* gene, which confers resistance to macrolides, were identified in plasmids of the three strains. The spread of antibiotic resistance genes (ARGs) is facilitated by mobile genetic elements (MGEs), which can be transferred from one strain to another, including integrons, transposons, and plasmids (Rozwadowski and Gawel, 2022). The RUTI strains UTI-1_774U, UTI-2_245U, and UTI-3_455U carried plasmids with mobilization genes (*mob*), transfer genes (*tra*), recombinase genes, and insertion sequence genes (IS) that facilitated their mobility; these events have been previously reported (Smillie et al., 2010). Additionally, the three strains showed a large group of small cryptic plasmids, identified by genomic homology studies between environmental strains, of veterinary importance, especially in uropathogenic clinical strains. The current function of these cryptic plasmids is unknown, and no previous reports have been found that further elucidate their importance. The evidence of the presence of repeated regions and the presence of guanine-cytosine islands suggests a possible regulatory function, and a greater number of studies are necessary to understand the role of these plasmids in RUTIs.

Furthermore, the three RUTI strains presented multiple genes for active efflux pumps and transporters that, together with the ARGs, may explain their MDR. Interestingly, the strain UTI-2_245U carried resistant genes to heavy metals. In recent years, heavy metal therapy has been proposed as an alternative to treatment in MDR strains (Wang et al., 2020). Sensitivity to bacteriophages used to treat MDR strains was also evaluated; the results indicated that the three RUTI strains were resistant to all bacteriophages evaluated (Supplementary Figure S9). Interestingly, in this work, the presence of an extrachromosomal prophage located in the UTI-2_245U strain was reported. The pEcoUTI2e plasmid belongs to the family of plasmids similar to bacteriophage the P1; within this context, this plasmid is an important source of MGEs, as has been previously reported (Billard-Pomares et al., 2014). However, in UTI-2_245U strains, this plasmid does not contain virulence or resistance factors, yet its potential for genomic plasticity is present. The phenotypic results of mobility showed the strains UTI-1_774U and UTI-3_455U as nonmotile and the strain UTI-2_245U (mobility zone 64 mm) as highly motile compared to the UPEC control strain CFT073 (mobility zone 32 mm). Genomic analysis of all flagellar genes showed that the three regions involved in flagellum biosynthesis were intact. However, minor differences were observed, including the loss of nonessential genes and the insertion of small ORFs of hypothetical proteins that do not alter the reading frame of the genes but that could be involved in the inhibition of flagellum formation (Fitzgerald et al., 2014).

The regulation of the flagellum could be influenced by the presence of a strong repressor of motility, *papX* (Simms and Mobley, 2008), present in strains UTI-1_774U and UTI-3_455U. The biofilm formation and haemolysis evaluation showed that the strains UTI-1_774U, UTI-2_245U, and UTI-3_455U were nonbiofilm producers and nonhemolytic in sheep blood. Biofilm formation is a multifactorial phenomenon that requires the presence of fimbriae, mainly type 1 fimbriae and Curli fimbriae, in addition to the flagellum, outer membrane proteins, and siderophores (Soto et al., 2007). Although Congo red uptake was evaluated as an indicator of curli generation and the strains showed fixation at 37°C (data not shown), biofilm formation could not be visualized under the conditions indicated in this study. As previously described, other relevant factors in adhesion and colonization were partially absent in the strains UTI-1_774U, UTI-2_245U, and UTI-3_455U; we suggest that the absence of one or more fitness factors causes the inhibition of biofilm formation (Qasemi et al., 2021).

Furthermore, haemolytic activity in sheep erythrocytes was not observed in the RUTI strains. The haemolysin-related genes are located on a genomic island adjacent to the pheV t-RNA in the UPEC reference strain CFT073; however, in strains UTI-1_774U and UTI-3_455U, the haemolysin region was wholly lost and replaced by the genes *irp1* and *irp2* (encodes ferric regulation genes), and *fyuA* (encodes an iron-inhibition outer membrane protein) in avian pathogenic *E. coli* strains (Tu et al., 2016). In the UTI-2_245U strain, the haemolysin genes were replaced by the *sigA* gene (encodes cytopathic protease in PAI-harboring *Shigella* sp., which contributes to intestinal fluid accumulation), and loss of the *sat* gene (encodes for a secreted proteolytic autotransporter proteins) that induces cell damage during enteroaggregative *E*. *coli* infection (Al-Hasani et al., 2001; Vieira et al., 2020). Additionally, the UTI-2_245U strain did not present the *irp1*, *irp2*, and *fyuA* genes, but the *chuA* gene, which encodes ferrochrome receptors, and the *kpsC* and *kpsS* genes, which are associated with capsule, were identified. Additionally, the genes *rfbA*, *rfbB*, and *rfbD* were identified that are not present in the reference strain CFT073 and that code for the biosynthesis of dTDP-rhamnose, which is the precursor of lipopolysaccharides of the cell wall. Finally, the *rpoS* gene encodes an RNA polymerase factor, which was not identified in strain CFT073, and the *sigA* gene, which is an autotransporter, was identified exclusively in strain UTI-2_245U. The siderophores and regulatory factors presented by strains UTI-1_774U, UTI-2_245U, and UTI-3_455U could contribute, in an important way, to chronicity in the urinary tract (Hunstad et al., 2005; Hryckowian and Welch, 2013; Gao et al., 2023). The absence of mobility, a critical factor in the rise of UPEC, to the upper urinary tract suggests an adaptation to the lower urinary tract. However, the UTI-2_245U strain showed increased mobility associated with its commensal origin, leading us to suggest that it is still in the adaptation process. To date, there is no effective vaccine that prevents RUTIs. In this context, regulation and effective decision-making against the indiscriminate use of antibiotics is urgently needed, given that alternatives to treatment in our strains are not viable options.

## 5 Limitations of the study

Although the study showed exciting results, the genotypic and phenotypic characterization was carried out only with 3 UPEC strains associated with RUTI, preventing us from generalizing the results and extrapolating them to other populations of recurrent strains. Under this same concept, it was tough to identify specific characteristics in the genomes of RUTI strains that could support the complete understanding of the persistence of the UPEC strains in the urinary tract. Another aspect of this study was that the UPEC strains were not recovered before the bladder biopsy, limiting the study of the evolution of the patient’s strains. In addition, a genomic comparison of the UPEC strains of the same patients was not displayed. However, the recovery of UPEC strains from bladder biopsies is an important aspect that must continue to be studied. Finally, database platforms must be updated continuously to generate more accurate knowledge that helps study recurrent bacterial populations.

## 6 Conclusion

We found that the decrease in classic colonization factors in our strains of RUTIs did not affect their capacity for adherence and invasion to human bladder HTB-5 cells, showing their adaptation to this ecological niche. We showed a profile of virulence, resistance, fitness factors, and high genetic diversity of RUTIs strains that could contribute to developing a recurrent infection in the urinary tract. This variability gives these strains a different arsenal of colonization and persistence strategies. Since the absence/presence of one or more virulence factors can influence the expression of other factors, we suggest that many of these systems may be redundant and cumulative. However, the expression of classical and highly virulent factors may allow UPEC strains to evade the acute immune response and promote a chronic infection in patients with a clinical predisposition to develop RUTIs. The results of this study did not allow us to identify a single factor responsible for the recurrent phenotype and genotype in UPEC strains associated with RUTIs.

## Data availability statement

The datasets presented in this study can be found in online repositories. The names of the repository/repositories and accession number(s) can be found in the article/Supplementary material.

## Ethics statement

Ethical approval was not required for the studies involving humans because as part a retrospective analysis, of storage remanents of biopsy samples, that are taken as part of the clinical diagnosis as otherwise be discarted, in this protocol the tissue samples was obtain after a appropiated diagnosis of mophological abnormalities, and medica consent includes the secundary use of tissue leftovers. Non genetic data was obtain of human samples, and all sensitive personal data was eliminated of this study. The studies were conducted in accordance with the local legislation and institutional requirements. The participants provided their written informed consent to participate in this study.

## Author contributions

MF-O: Formal analysis, Investigation, Methodology, Writing – original draft, Writing – review & editing, Conceptualization, Data curation, Visualization. SO: Conceptualization, Formal analysis, Funding acquisition, Resources, Supervision, Writing – original draft. AC-C: Funding acquisition, Resources, Supervision, Writing – review & editing. RC-T: Investigation, Methodology, Resources, Writing – review & editing. EM-P: Formal analysis, Investigation, Methodology, Software, Visualization, Writing – review & editing. DR-B: Investigation, Methodology, Resources, Visualization, Writing – review & editing. SZ-V: Methodology, Writing – review & editing. RH-C: Funding acquisition, Resources, Writing – review & editing. MF-E: Resources, Writing – review & editing. JA-G: Resources, Writing – review & editing. DV: Conceptualization, Resources, Writing – review & editing. JX-C: Conceptualization, Formal analysis, Funding acquisition, Investigation, Methodology, Project administration, Resources, Software, Supervision, Writing – original draft, Writing – review & editing.

## Footnotes

^1^ www.graphpad.com

^2^ https://github.com/rrwick/Unicycler

^3^ https://www.ncbi.nlm.nih.gov/bioproject/610084

^4^ https://www.ncbi.nlm.nih.gov/genome/annotation_prok/

^5^ https://www.ncbi.nlm.nih.gov/genome/annotation_euk/

^6^ https://www.geneious.com

^7^from Dotmatics; available at snapgene.com

^8^ https://www.ebi.ac.uk/interpro/search/sequence/

^9^ https://www.ncbi.nlm.nih.gov/

^10^ https://www.bv-brc.org/

^11^ https://www.bv-brc.org/app/PhylogeneticTree

^12^ https://itol.embl.de/

^13^ https://inkscape.org/

^14^ https://cge.food.dtu.dk/services/Virulen

^15^ https://tool2-mml.sjtu.edu.cn/VRprofile/VRprofile.php

^16^ https://bioinfo-mml.sjtu.edu.cn/oriTfinder/

^17^ https://www.bv-brc.org/

^18^ https://cge.cbs.dtu.dk/services/ResFinder/

^19^CARD, https://card.mcmaster.ca/home

^20^ https://www.uniprot.org/

^21^ https://www.ncbi.nlm.nih.gov/

^22^ https://www.pathogenomics.sfu.ca/islandviewer/

^23^ https://phaster.ca/

^24^ https://www.ridom.de/u/E._coli_CGE_SerotypeFinder.html

^25^https://pubmlst.org/escherichia/; accessed on 13 October 2021.
