# Supplementary figures and images for: Comparative genomic analysis of uropathogenic Escherichia coli strains from women with recurrent urinary tract infection
Source: Front Microbiol. 2024 Jan 24;14:1340427. doi: 10.3389/fmicb.2023.1340427 (PMC10848155; doi:10.3389/fmicb.2023.1340427)

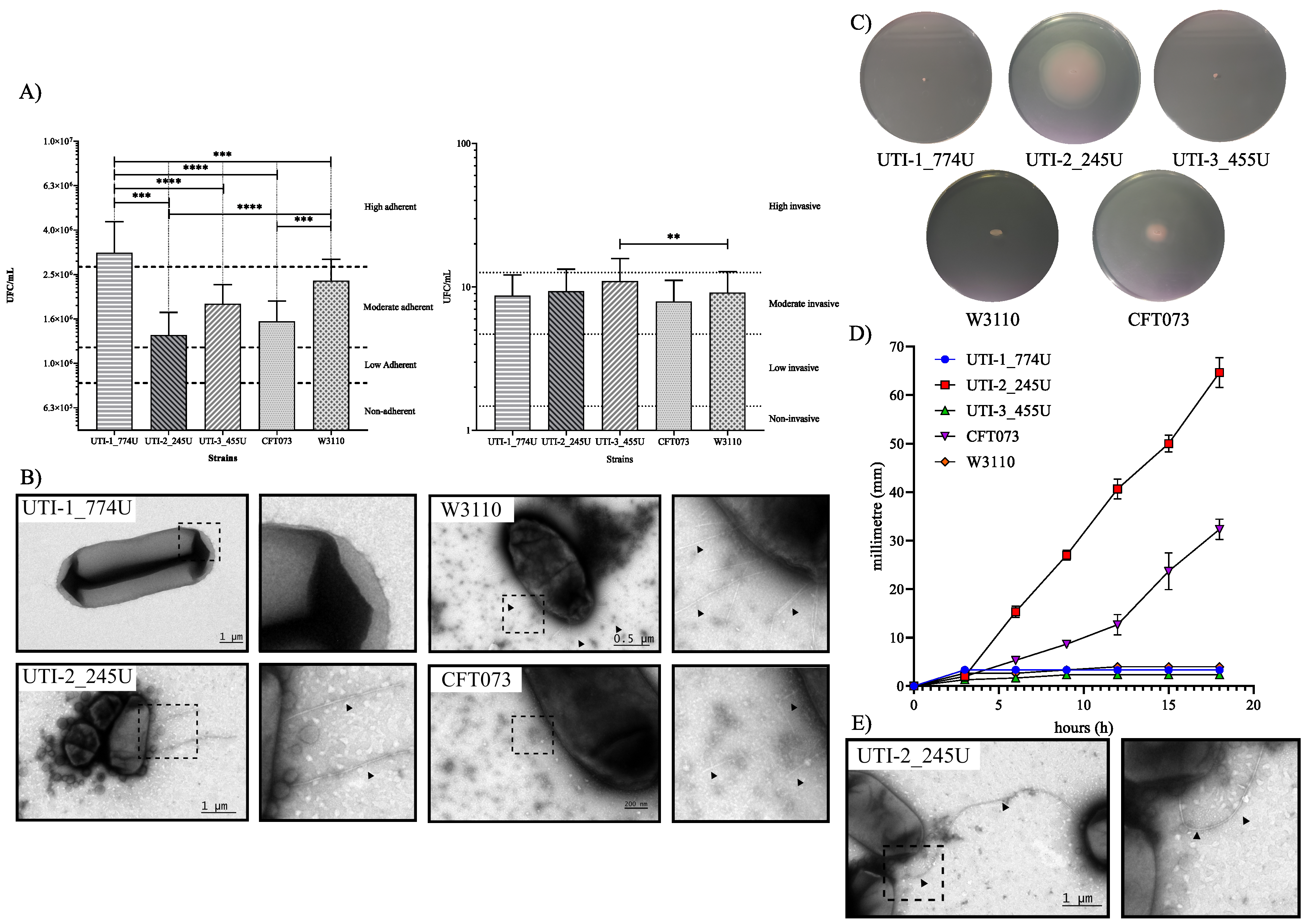

Supplement: Supplementary file 2 [file Figure_2.TIFF]

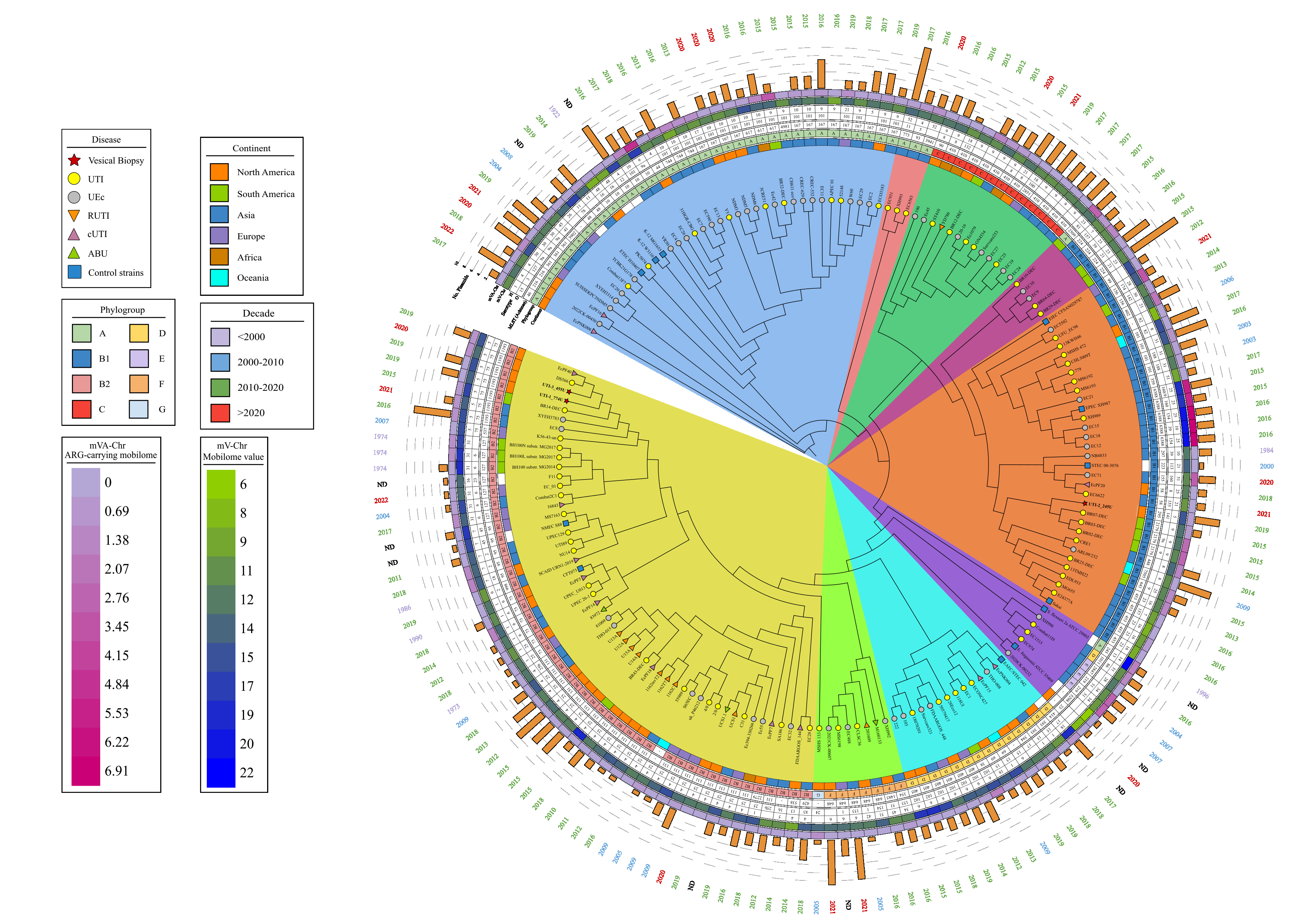

Supplement: Supplementary file 3 [file Figure_3.TIFF]

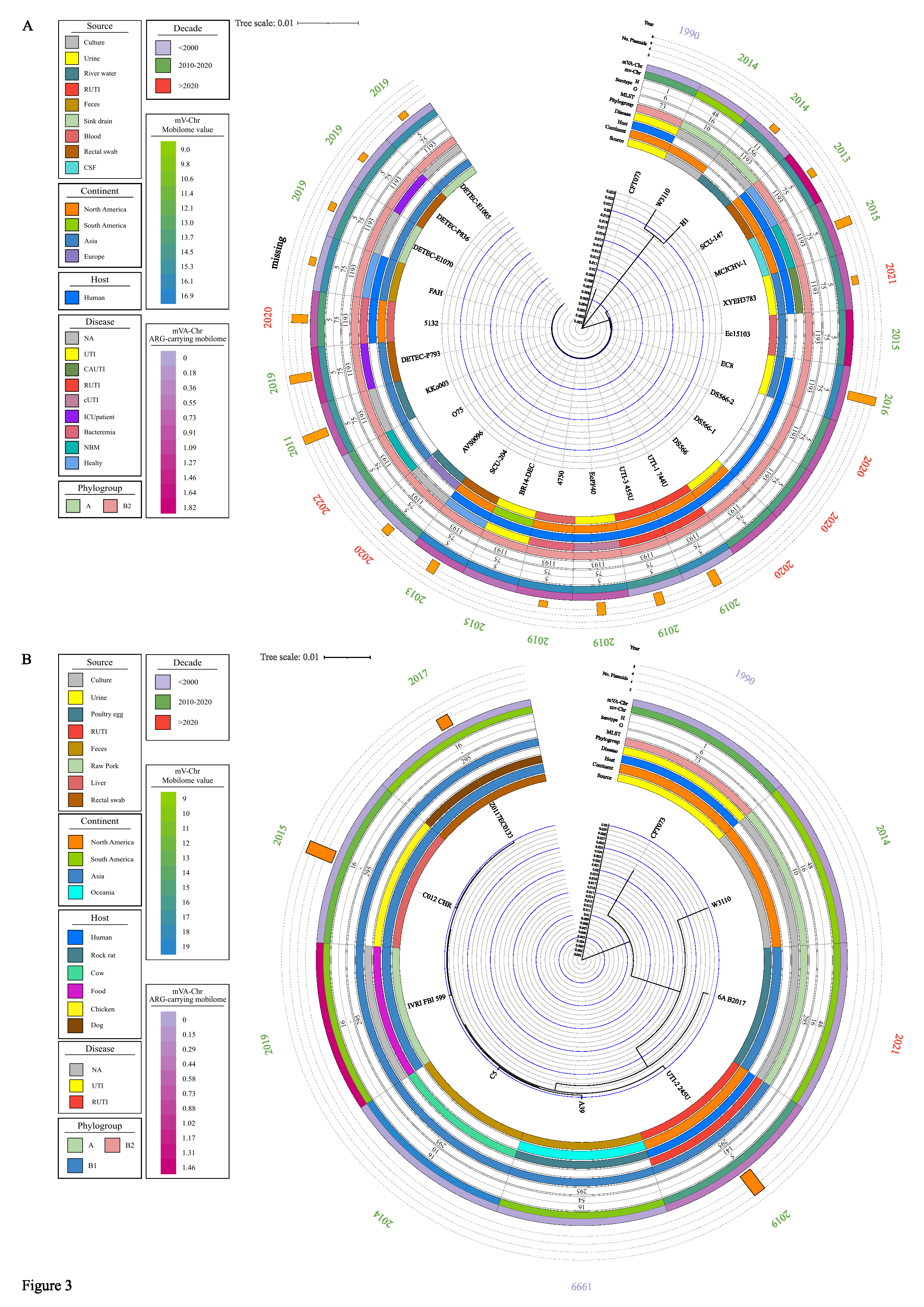

Supplement: Supplementary file 4 [file Figure_4.TIFF]

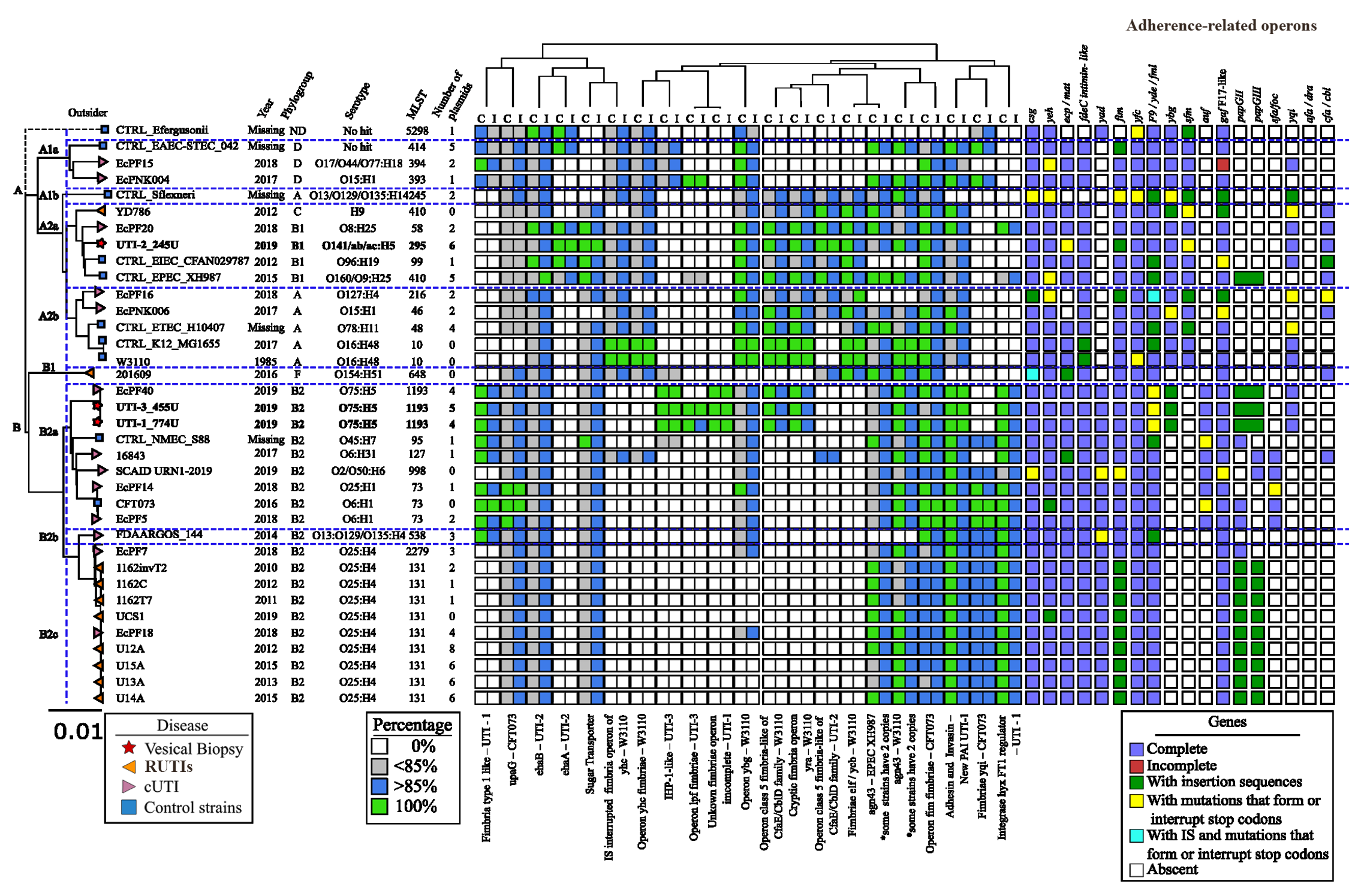

Supplement: Supplementary file 5 [file Figure_5.TIFF]

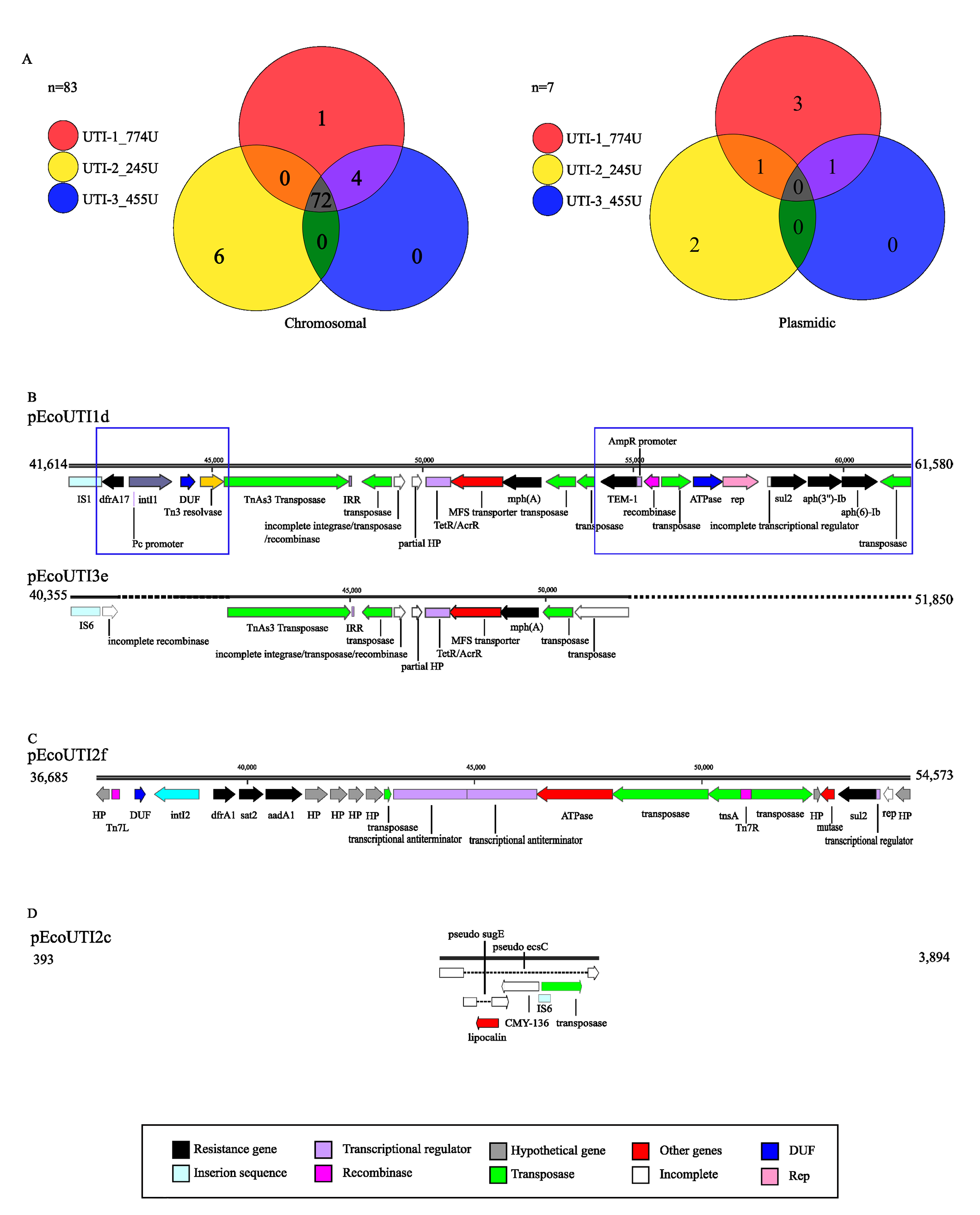

Supplement: Supplementary file 6 [file Figure_6.TIFF]
