# Supplementary material for: Comparative genomic analysis of uropathogenic Escherichia coli strains from women with recurrent urinary tract infection
Source: Front Microbiol. 2024 Jan 24;14:1340427. doi: 10.3389/fmicb.2023.1340427 (PMC10848155; doi:10.3389/fmicb.2023.1340427)
Supplement: Supplementary file 7 [file Data_Sheet_1.zip › Supplementary Figures.pdf]

Timeline of evolution of RUTIs in patients

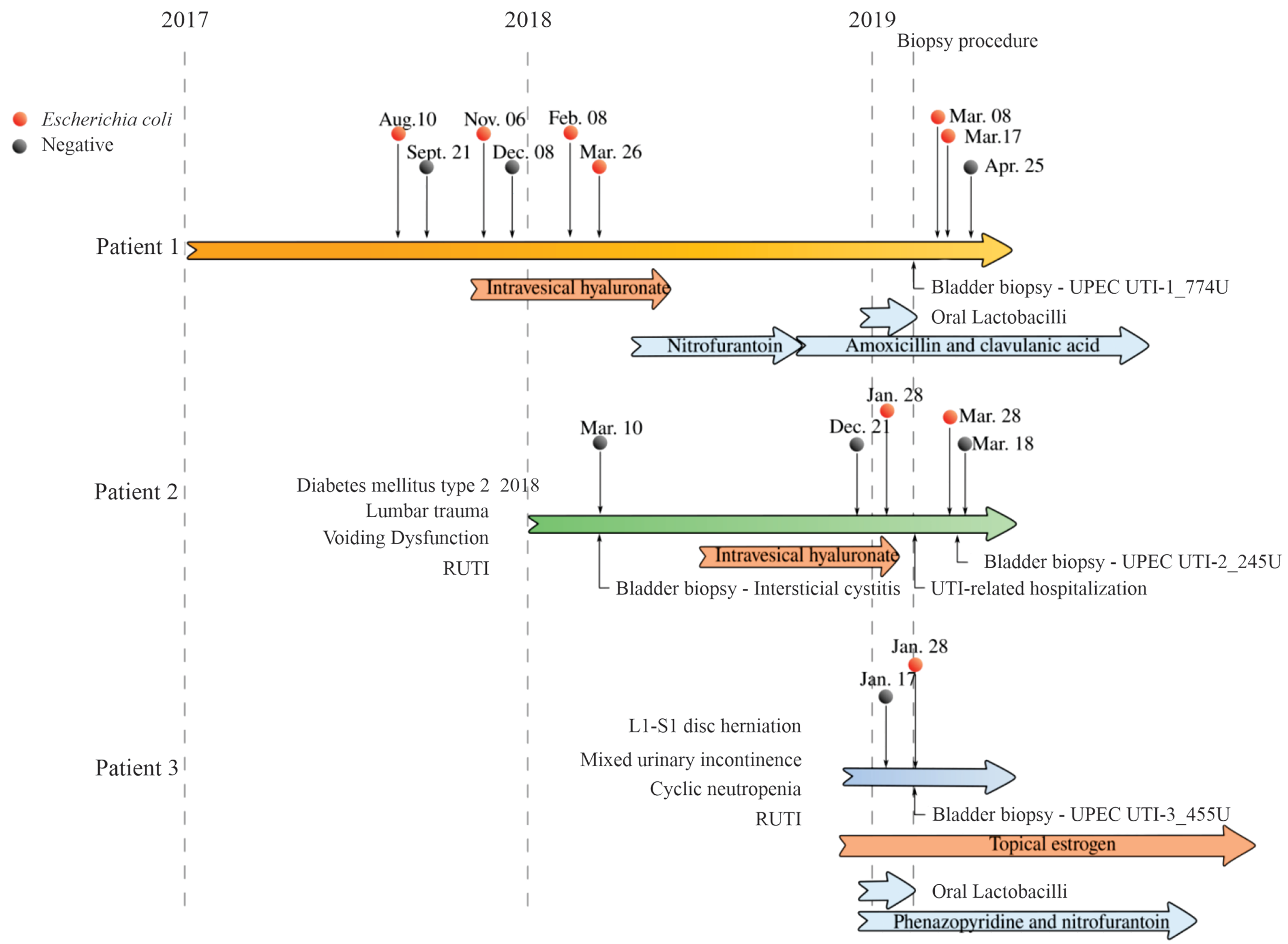

Hemolytic activity of RUTI-associated UPEC strains

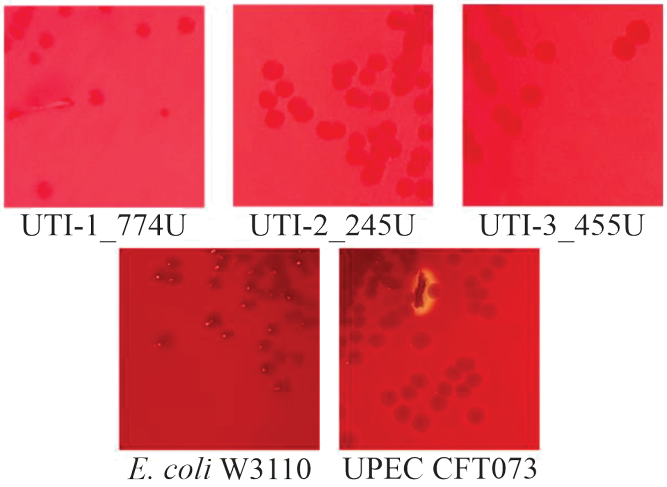

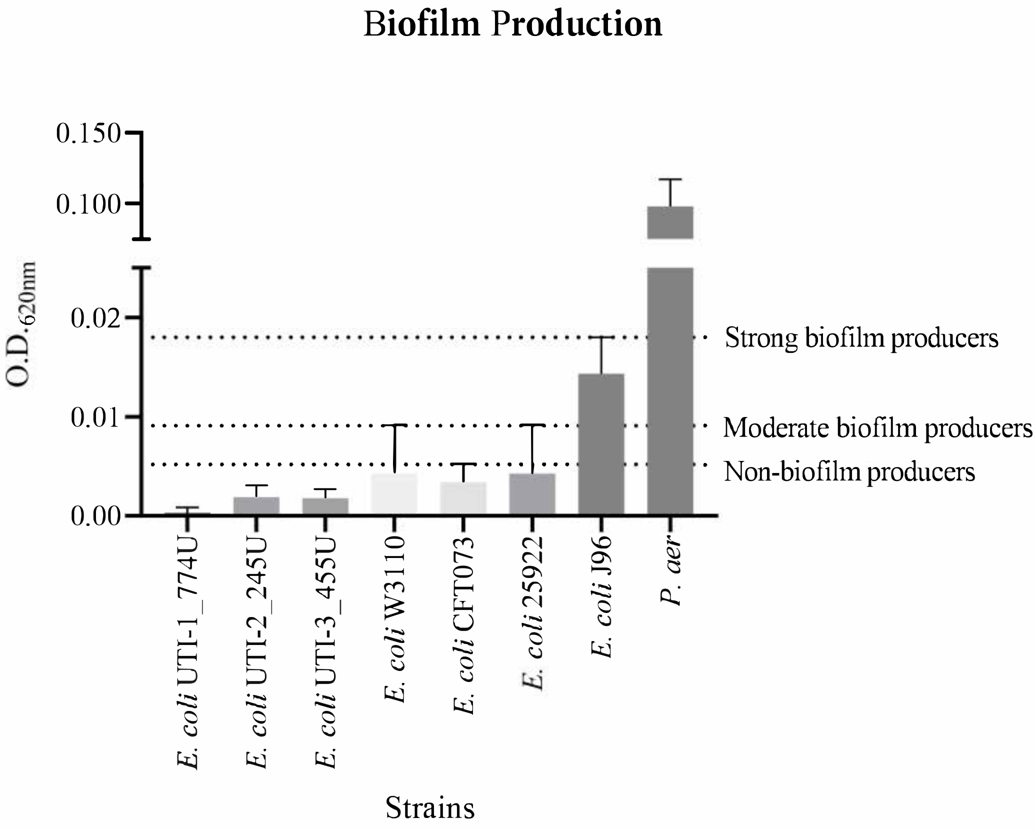

Biofilm Production
0.150
0.100
0.02
0.01
0.00
O.D.620nm
Strong biofilm producers
Moderate biofilm producers
Non-biofilm producers
E. coli UTI-1_774U
E. coli UTI-2_245U
E. coli UTI-3_455U
E. coli W3110
E. coli CFT073
E. coli 25922
E. coli J96
P. aer
Strains

CFT073 Pathogenicity Islands Loss in RUTIs

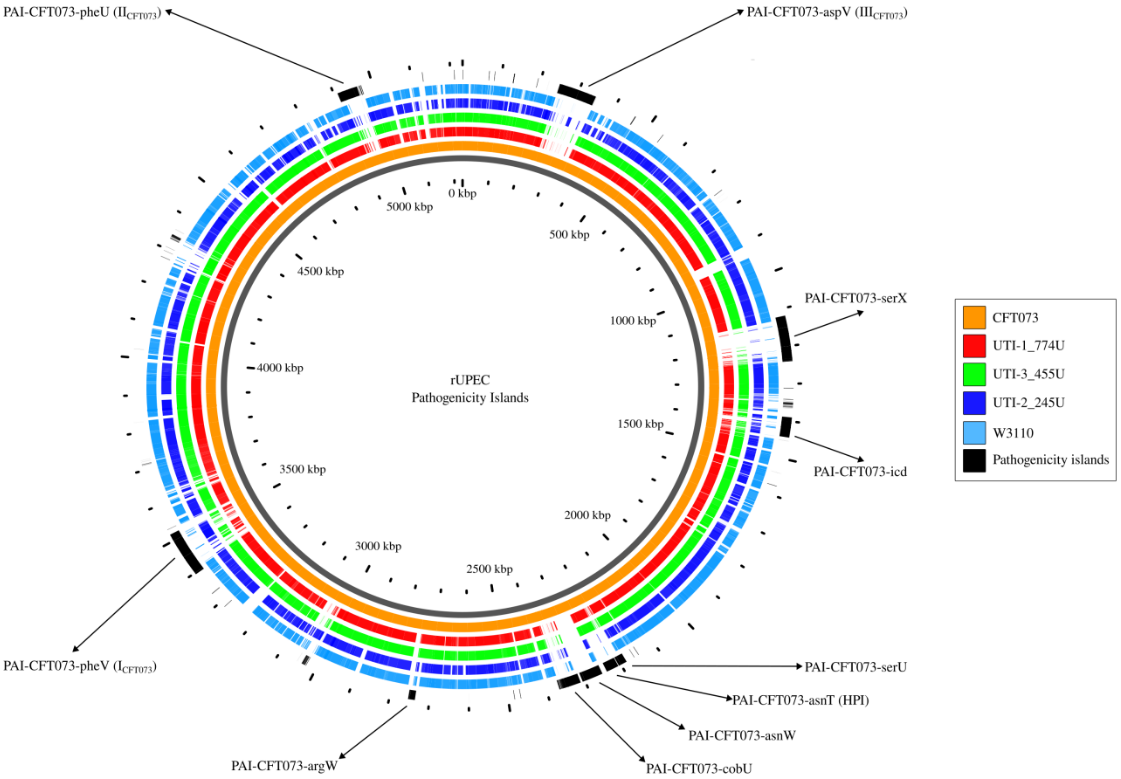

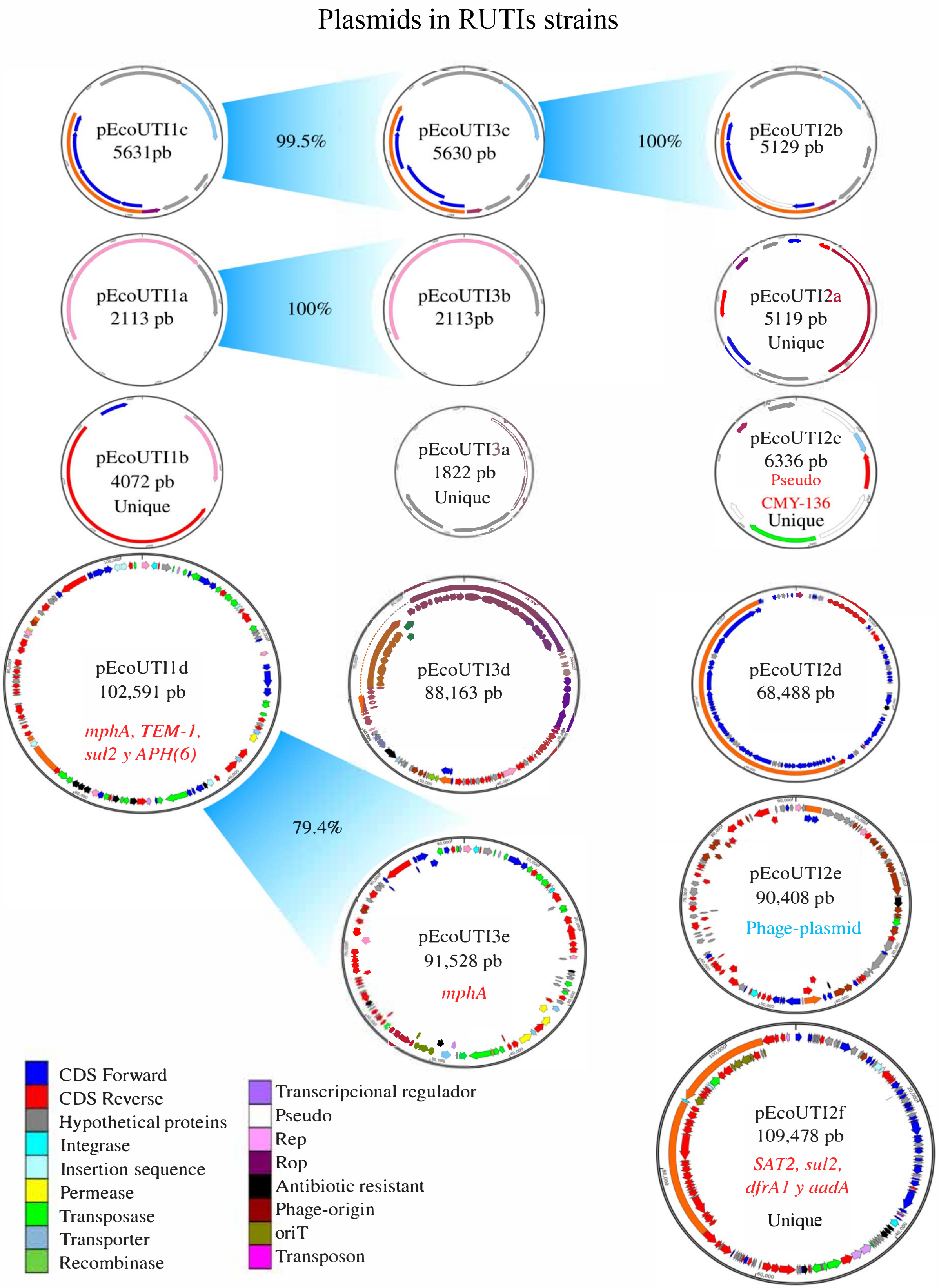
Plasmids in RUTIs strains
pEcoUTI1c
5631pb
99.5%
pEcoUTI3c
5630 pb
100%
pEcoUTI2b
5129 pb
pEcoUTI1a
2113 pb
100%
pEcoUTI3b
2113pb
pEcoUTI2a
5119 pb
Unique
pEcoUTI1b
4072 pb
Unique
pEcoUTI3a
1822 pb
Unique
pEcoUTI2c
6336 pb
Pseudo
CMY-136
Unique
pEcoUTI1d
102,591 pb
mphA, TEM-1,
sul2 y APH(6)
pEcoUTI3d
88,163 pb
pEcoUTI2d
68,488 pb
79.4%
pEcoUTI3e
91,528 pb
mphA
pEcoUTI2e
90,408 pb
Phage-plasmid
pEcoUTI2f
109,478 pb
SAT2, sul2,
dfrA1 y aadA
Unique
CDS Forward
CDS Reverse
Hypothetical proteins
Integrase
Insertion sequence
Permease
Transposase
Transporter
Recombinase
Transcripcional regulador
Pseudo
Rep
Rop
Antibiotic resistant
Phage-origin
oriT
Transposon

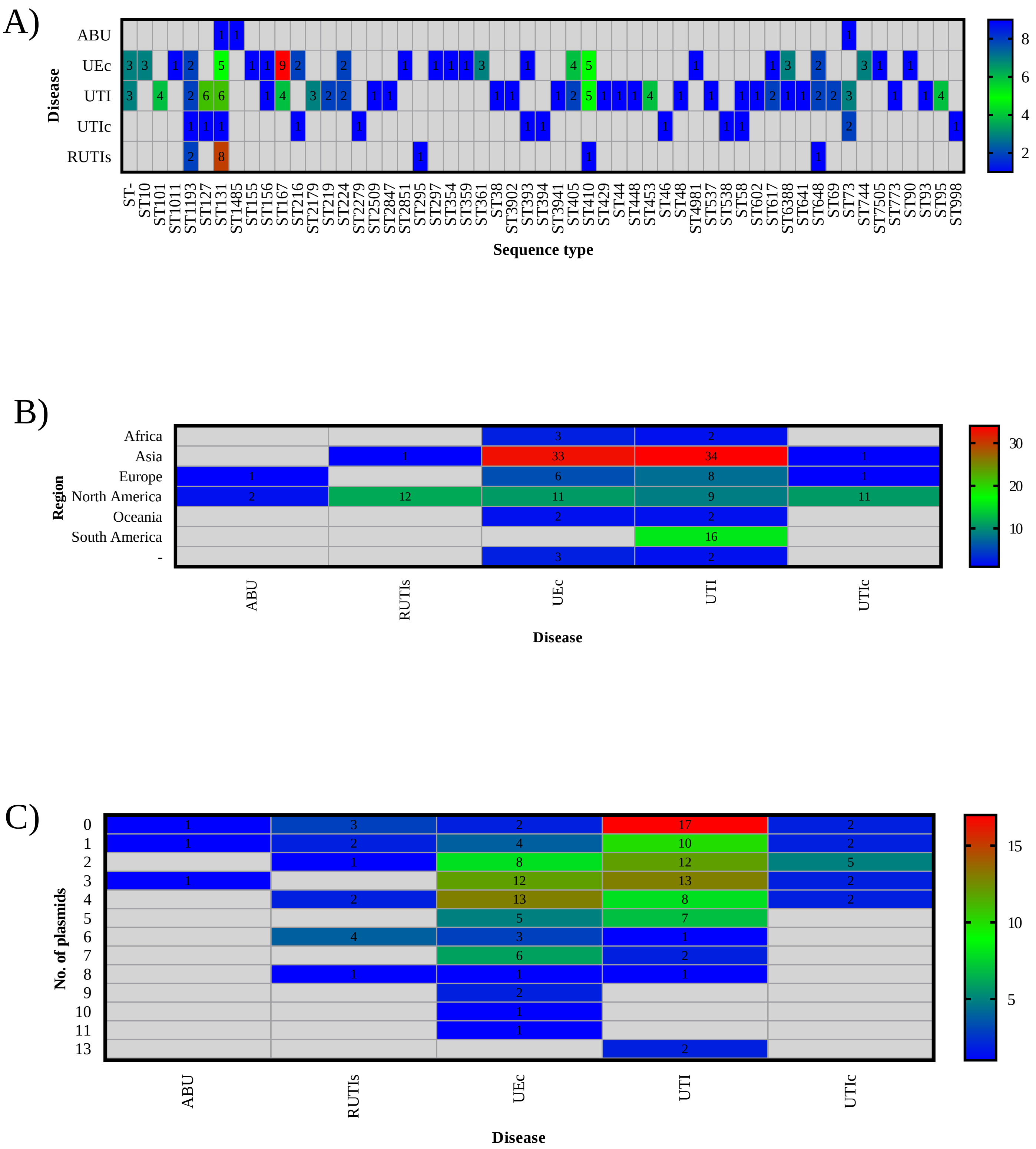
A)
Disease
ABU
UEc
UTI
UTIc
RUTIs
Sequence type
ST-
ST10
ST101
ST1011
ST1193
ST127
ST131
ST1485
ST155
ST156
ST167
ST216
ST2179
ST219
ST224
ST2279
ST2509
ST2847
ST2851
ST295
ST297
ST354
ST359
ST361
ST38
ST3902
ST393
ST394
ST3941
ST405
ST410
ST429
ST44
ST448
ST453
ST46
ST48
ST4981
ST537
ST538
ST58
ST602
ST617
ST6388
ST641
ST648
ST69
ST73
ST744
ST7505
ST773
ST90
ST93
ST95
ST998
B)
Region
Africa
Asia
Europe
North America
Oceania
South America
-
ABU
RUTIs
UEc
UTI
UTIc
Disease
C)
No. of plasmids
ABU
RUTIs
UEc
UTI
UTIc
Disease

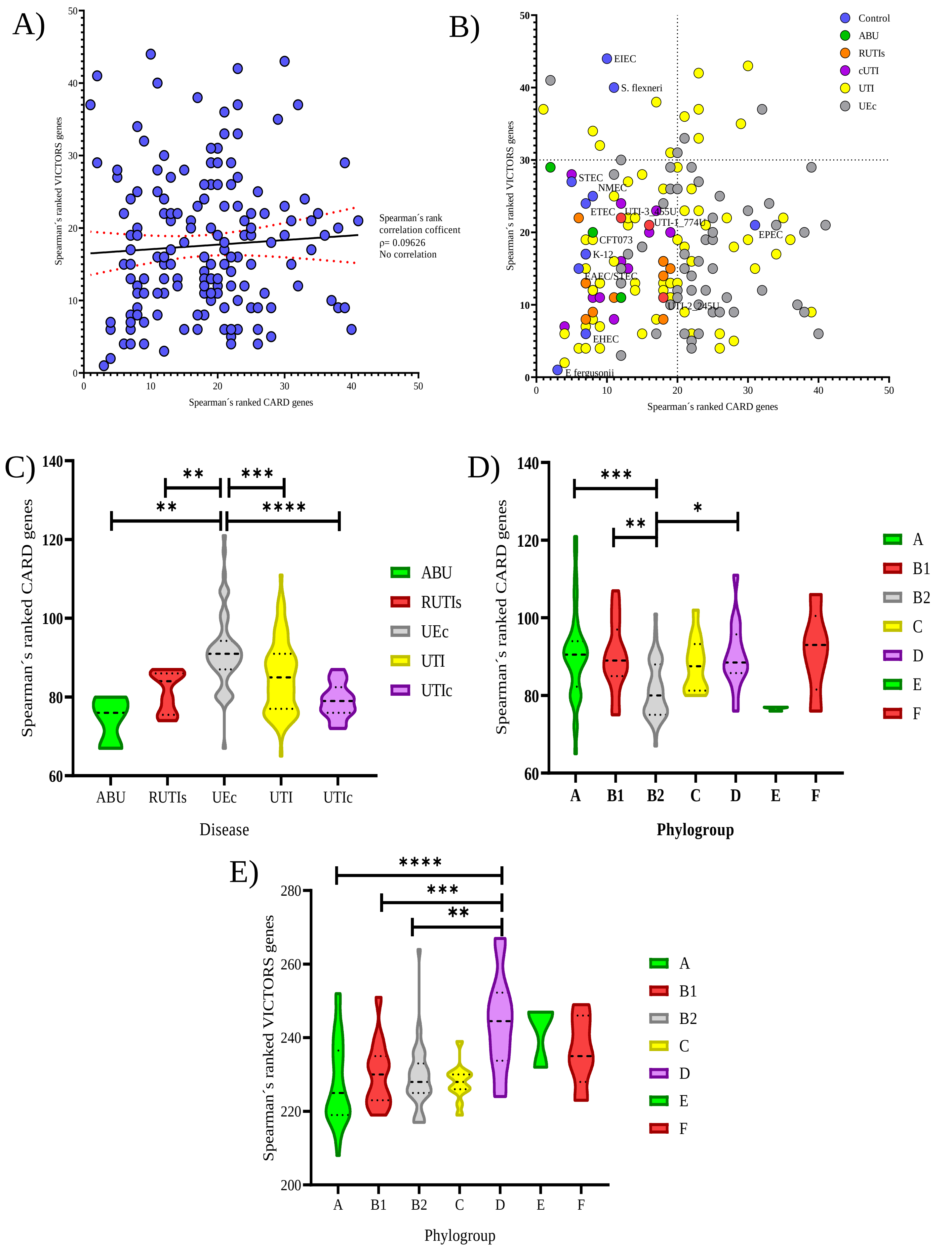

A)
Spearman´s ranked VICTORS genes
Spearman´s ranked CARD genes
Spearman´s rank correlation cofficent
ρ= 0.09626
No correlation
B)
Control
ABU
RUTIs
cUTI
UTI
UEc
EIEC
S. flexneri
STEC
NMEC
ETEC
UTI-3_455U
UTI-1_774U
CFT073
K-12
EAEC/STEC
UTI-2_245U
EHEC
E.fergusonii
EPEC
Spearman´s ranked VICTORS genes
Spearman´s ranked CARD genes
C)
Spearman´s ranked CARD genes
ABU
RUTIs
UEc
UTI
UTIc
Disease
D)
Spearman´s ranked CARD genes
A
B1
B2
C
D
E
F
Phylogroup
E)
Spearman´s ranked VICTORS genes
A
B1
B2
C
D
E
F
Phylogroup

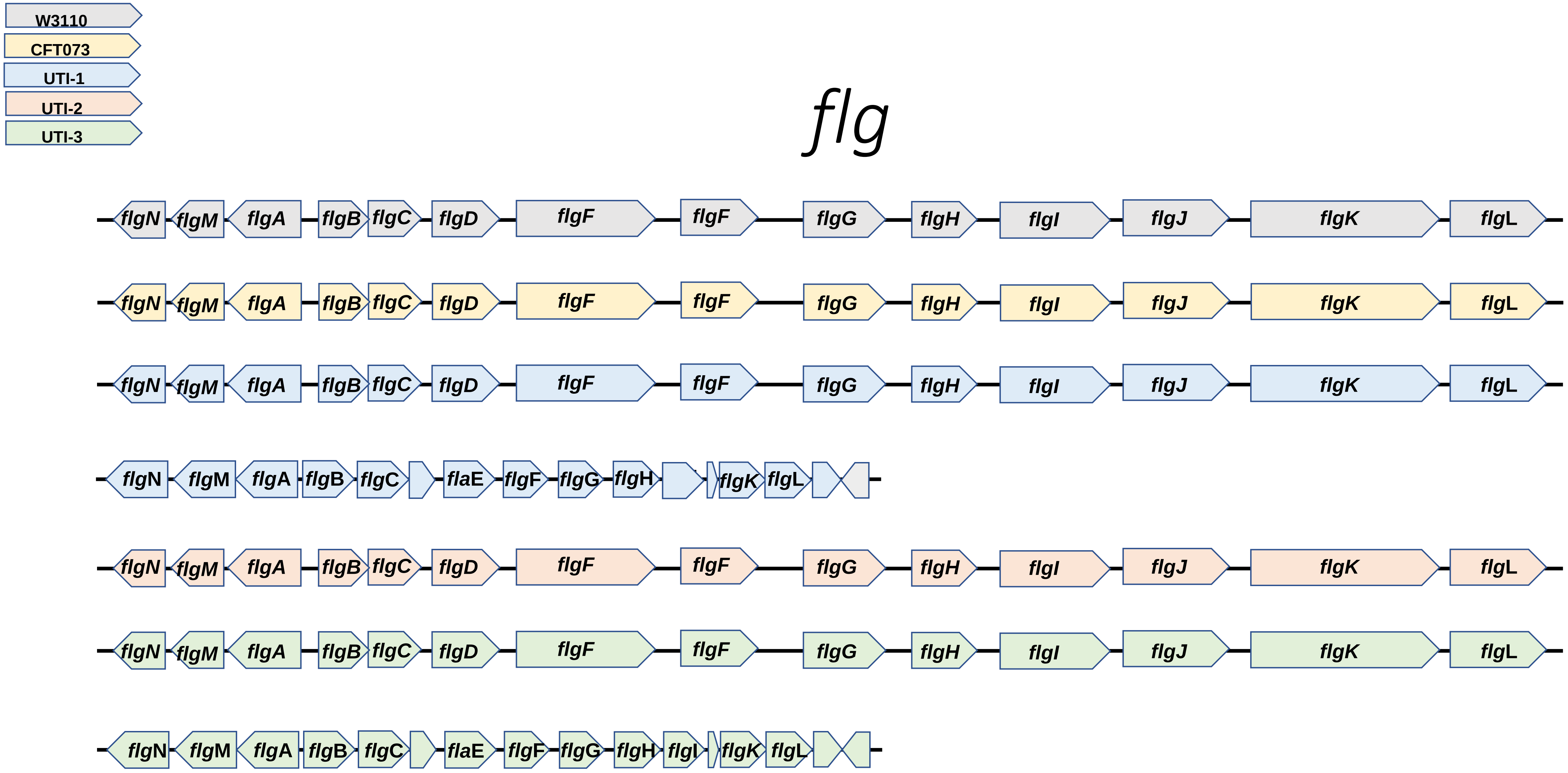
W3110
CFT073
UTI-1
UTI-2
UTI-3
flg
flgN flgM flgA flgB flgC flgD flgF flgF flgG flgH flgI flgJ flgK flgL
flgN flgM flgA flgB flgC flgD flgF flgF flgG flgH flgI flgJ flgK flgL
flgN flgM flgA flgB flgC flgD flgF flgF flgG flgH flgI flgJ flgK flgL
flgN flgM flgA flgB flgC flaE flgF flgG flgH flgK flgL
flgN flgM flgA flgB flgC flgD flgF flgF flgG flgH flgI flgJ flgK flgL
flgN flgM flgA flgB flgC flgD flgF flgF flgG flgH flgI flgJ flgK flgL
flgN flgM flgA flgB flgC flaE flgF flgG flgH flgI flgK flgL

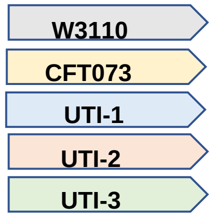
W3110
CFT073
UTI-1
UTI-2
UTI-3

flh

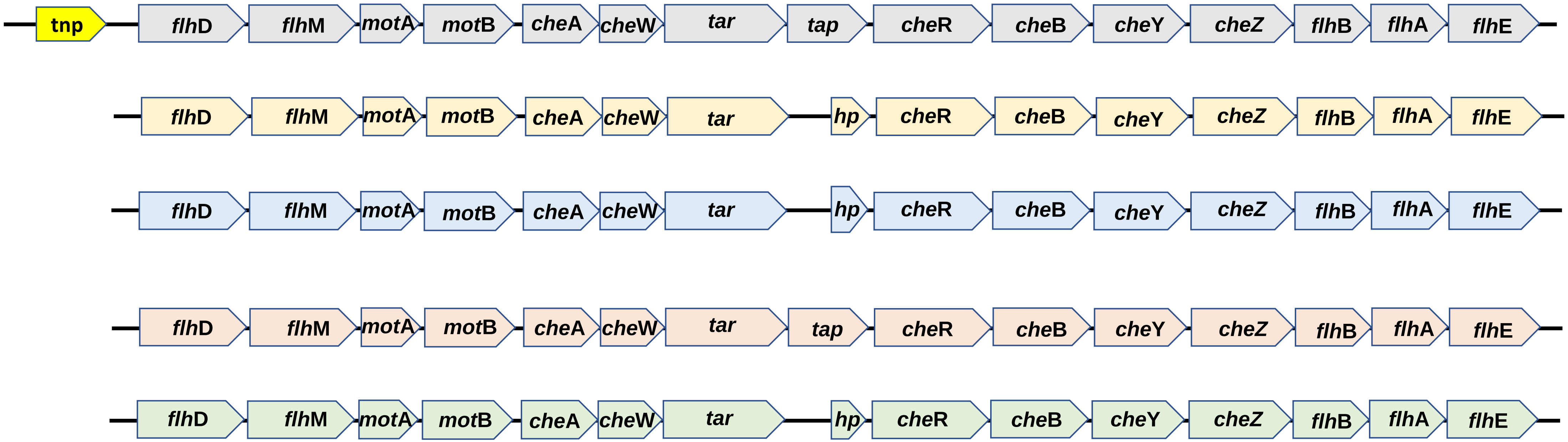
tnp flhD flhM motA motB cheA cheW tar tap cheR cheB cheY cheZ flhB flhA flhE
flhD flhM motA motB cheA cheW tar hp cheR cheB cheY cheZ flhB flhA flhE
flhD flhM motA motB cheA cheW tar hp cheR cheB cheY cheZ flhB flhA flhE
flhD flhM motA motB cheA cheW tar tap cheR cheB cheY cheZ flhB flhA flhE
flhD flhM motA motB cheA cheW tar hp cheR cheB cheY cheZ flhB flhA flhE

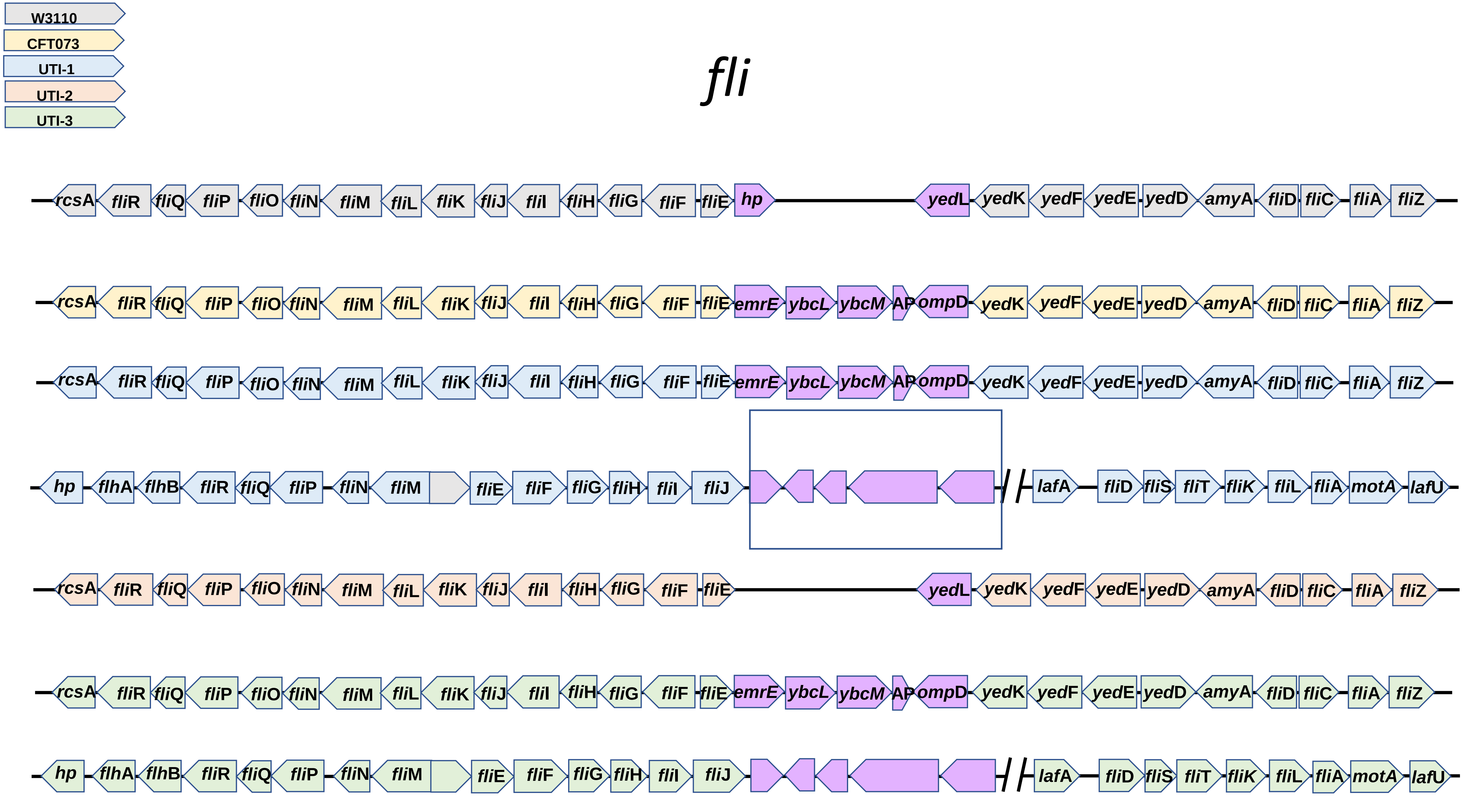
W3110
CFT073
UTI-1
UTI-2
UTI-3
fli
rcsA fliR fliQ fliP fliO fliN fliM fliL fliK fliJ fliI fliH fliG fliF fliE hp yedL yedK yedF yedE yedD amyA fliD fliC fliA fliZ
rcsA fliR fliQ fliP fliO fliN fliM fliL fliK fliJ fliI fliH fliG fliF fliE emrE ybcL ybcM AP ompD yedK yedF yedE yedD amyA fliD fliC fliA fliZ
rcsA fliR fliQ fliP fliO fliN fliM fliL fliK fliJ fliI fliH fliG fliF fliE emrE ybcL ybcM AP ompD yedK yedF yedE yedD amyA fliD fliC fliA fliZ
hp flhA flhB fliR fliQ fliP fliN fliM fliE fliF fliG fliH fliI fliJ lafA fliD fliS fliT fliK fliL fliA motA lafU
rcsA fliR fliQ fliP fliO fliN fliM fliL fliK fliJ fliI fliH fliG fliF fliE yedL yedK yedF yedE yedD amyA fliD fliC fliA fliZ
rcsA fliR fliQ fliP fliO fliN fliM fliL fliK fliJ fliI fliH fliG fliF fliE emrE ybcL ybcM AP ompD yedK yedF yedE yedD amyA fliD fliC fliA fliZ
hp flhA flhB fliR fliQ fliP fliN fliM fliE fliF fliG fliH fliI fliJ lafA fliD fliS fliT fliK fliL fliA motA lafU

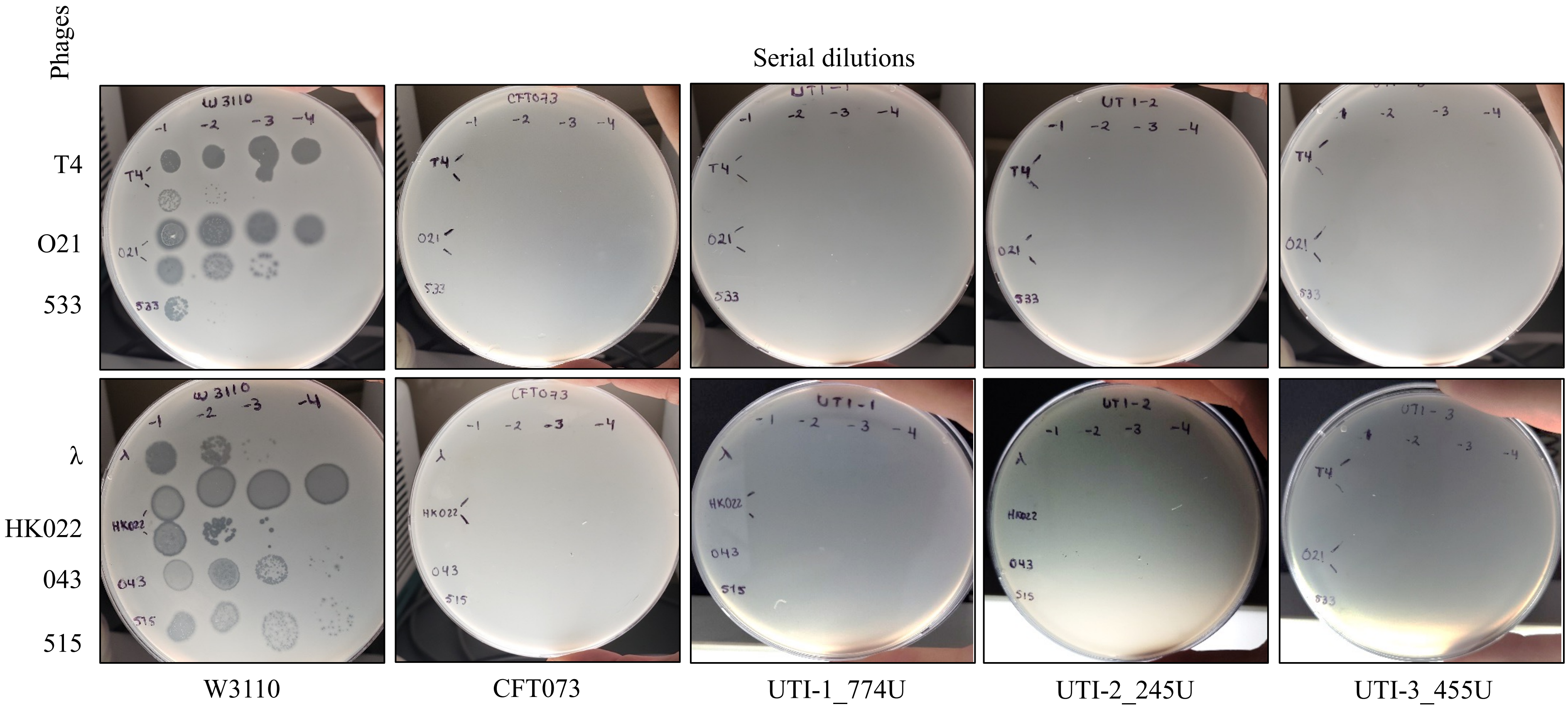

Bacteriophages sensitivity
