# Supplementary material for: Comparative genomic analysis of uropathogenic Escherichia coli strains from women with recurrent urinary tract infection
Source: Front Microbiol. 2024 Jan 24;14:1340427. doi: 10.3389/fmicb.2023.1340427 (PMC10848155; doi:10.3389/fmicb.2023.1340427)
Supplement: Supplementary file 7 [file Data_Sheet_1.zip › Supplementary Table S1.DOCX]

Supplementary Table 1. Main virulence genes associated to recurrent UPEC strains involved to process of RUTIs according to clinical data.

| **Virulence Factor** | **Operon** | **UPEC CFT073** | ***E. coli* W3110** | **UTI-1_774U** | **UTI-2_245U** | **UTI-3_455U** |
| --- | --- | --- | --- | --- | --- | --- |
| **Fimbriae related to adherence/invasion/biofilm formation** | | | | | | |
| Curli | *Csg* | 1 | 1 | 1 | 1 | 1 |
| Yeh | *Yeh* | 0 | 1 | 1 | 1 | 1 |
| *E*. *coli* common pili | *ecp / mat* | 1 | 1 | 1 | 0 | 1 |
| Type 1 | *Fim* | 1 | 1 | 1 | 0 | 1 |
| Intimin-like | *Fde* | 1 | 0 | 1 | 1 | 1 |
| Trimeric autotransporter adhesin UpaG | *upaG* | 0 | 1 | 1 | 1 | 1 |
| Yad | *Yad* | 1 | 0 | 1 | 0 | 1 |
| Yfc | *Yfc* | 1 | 0 | 1 | 1 | 1 |
| F9 | *yde / fml* | 0 | 1 | 0 | 1 | 0 |
| Ybg | *Ybg* | 0 | 1 | 0 | 1 | 0 |
| Type 1 of *Salmonella* | *Sfm* | 0 | 1 | 0 | 0 | 0 |
| UTI-associated fimbrial operon | *Auf* | 0 | 0 | 1 | 0 | 1 |
| G | *Gaf* | 1 | 1 | 1 | 1 | 1 |
| P | *Pap* | 1 | 0 | 0 | 0 | 0 |
| S/F1C | *sfa / foc* | 1 | 0 | 0 | 0 | 0 |
| Yqi | *Yqi* | 0 | 1 | 1 | 1 | 1 |
| Dr/afimbrial adhesins "afa" | *afa / dra* | 0 | 0 | 0 | 0 | 0 |
| Lpf | *Lpf* | 0 | 0 | 0 | 1 | 1 |
| Type V pili | *Pil* | 0 | 1 | 0 | 1 | 1 |
| Outer membrane protein T | *Omp* | 1 | 1 | 1 | 1 | 1 |
| Toxin co-regulated pilus | *Tcp* | 1 | 0 | 0 | 0 | 0 |
| CFA/I | *cfa / cbl* | 0 | 0 | 0 | 1 | 0 |
| **Exotoxins** | | | | | | |
| α-hemolysin | *Hly* | 1 | 0 | 0 | 0 | 0 |
| Cytotoxic necrotizing factor 1 | *Cnf* | 1 | 0 | 0 | 0 | 0 |
| EAST1 | *Ast* | 0 | 0 | 0 | 0 | 0 |
| Citolethal distending | *cdt* | 0 | 0 | 0 | 0 | 0 |
| Colibactin | *Clb* | 1 | 0 | 0 | 0 | 0 |
| **Metabolism and nutrition** | | | | | | |
| Enterobactin | *ent, fes y dep* | 1 | 1 | 1 | 1 | 1 |
| Aerobactin | *Iut* | 1 | 0 | 1 | 1 | 1 |
| Sideróforo Salmochelina | *Iro* | 1 | 0 | 0 | 0 | 0 |
| Chu (grupo heme uptake) | *Chu* | 1 | 0 | 1 | 0 | 1 |
| Yersiniabactina | *ybt / irp* | 1 | 0 | 1 | 0 | 1 |
| **Antitoxin – toxin** | | | | | | |
| Antigen 43 | *agn43* | 1 | 1 | 1 | 1 | 1 |
| Secreted autotransporter | *sat* | 1 | 0 | 1 | 0 | 1 |
| Vacuolating autotransporter | *vat* | 1 | 0 | 1 | 0 | 1 |
| Temperature sensitive hemmagglutinin | *tsh* | 1 | 0 | 1 | 0 | 1 |
| Serin protease autotransporter | *pic* | 1 | 0 | 0 | 0 | 0 |
| **Miscellaneous** | | | | | | |
| Capsule | *kps/neu* | 1 | 1 | 1 | 0 | 1 |
| iron-responsive element | *ireA* | 1 | 0 | 0 | 0 | 0 |
| Flagella | *flg, flh* and *fliC* | 1 | 1 | 1 | 1 | 1 |
