# Supplementary material for: Comparative genomic analysis of uropathogenic Escherichia coli strains from women with recurrent urinary tract infection
Source: Front Microbiol. 2024 Jan 24;14:1340427. doi: 10.3389/fmicb.2023.1340427 (PMC10848155; doi:10.3389/fmicb.2023.1340427)
Supplement: Supplementary file 7 [file Data_Sheet_1.zip › Supplementary Table S2.DOCX]

| **Supplementary Table 2**. General data of the assembled genomes from recurrent UPEC strains. | | | |
| --- | --- | --- | --- |
|  | UTI-1_774U | UTI-2_245U | UTI-3_455U |
| Organism name | *Escherichia coli* | | |
| Taxonomy check | Yes | | |
| BioSample | SAMN14277526 | SAMN14277527 | SAMN14277528 |
| BioProject | PRJNA610084 | | |
| Submitter | Universidad Nacional Autónoma de México | | |
| Date | 03/08/2021 | | |
| Assembly type | NA | | |
| Assembly level | Complete Genome | | |
| Genome representation | Full | | |
| GenBank^®^ assembly accession | GCA_019443685.1 | GCA_023376095.1 | GCA_019443605.1 |
| RefSeq assembly accession | GCF_019443685.1 | GCF_023376095.1 | GCF_019443605.1 |
| Assembly method | Unicycler v. v0.4.1 | | |
| Genome coverage | 189.523x | 208.905x | 286.563x |
| Sequencing technology | Illumina HiSeq^®^; Oxford Nanopore MinION^®^ | | |

RefSeq: NCBI Reference Sequence Database; NA: not apply.
