# Supplementary material for: Comparative genomic analysis of uropathogenic Escherichia coli strains from women with recurrent urinary tract infection
Source: Front Microbiol. 2024 Jan 24;14:1340427. doi: 10.3389/fmicb.2023.1340427 (PMC10848155; doi:10.3389/fmicb.2023.1340427)
Supplement: Supplementary file 7 [file Data_Sheet_1.zip › Supplementary Table S3.DOCX]

| Supplementary Table 3. CRISPR arrays found in the genomes of the recurrent UPEC strains from this study. | | |
| --- | --- | --- |
| Repeat | **Spacer** | **BLAST prediction** |
| Repeat region 1: UTI-1_774U [2136831:2137652] forward | | |
| CGCGTCTTATCAGGCCTACGAGTTCGGTGCTGTGTAGGTCGGATAAGGCGTTCA | TGCCGCATCCGACAATAACAGCATTGCCTGATGCGACGCTTG | Ec, Ec O157:H7. |
| CGCGTCTTATCAGGCCTACGAGTTCAGTGCTGTGTAGGTCGGATAAGGCGTTCA |  |  |
| Repeat region 2: UTI-1_774U [1608639:1608786] forward | | |
| GTTCACTGCCGTACAGGCAGCTTAGAAA | GCACACGTCGAGCTGGTGGGGATTAATGCTGG | Ec, Ec 536, Ec O1:HNT, Ec SMS-3-5, Sf*,* Eh*.* |
| GTTCACTGCCGTACAGGCAGCTTAGAAA | TTGCCGCGGATCCTGTCTGCCAATAATGACAA | Ec, Em, Ec O99:H6, Ec O25:H4. |
| GTTCACTGCCGTACAGGCAGATAAAATG |  |  |
| Repeat region 3: UTI-1_774U [3094136:3094262] forward | | |
| TTTGTAGGCCTGATAAGACGCGCCAGCGTCGCATCAGGC | TCCGGGTGCCGGATGCAGCGTGAACGCCTTATCCGGCCTACGGCTCGGA | Ec, Sf, Ss. |
| TTTGTAGGCCTGATAAGACGCGCCAGCGTCGCATCAGGC |  |  |
| Repeat region 4: UTI-1_774U [4910221:4910353] ND Possible forward | | |
| ACGCTGCCGCGTCTTATCGGGCCTACAAAAGTTCTGAACCGT | GGGCCGGATAAGGCGTTCACGCCGTATCCGGCATACAGTGCCCGATGCG | Ec, Ec O4:H5, Ec O84:H7, Sf, Eh. |
| ACGCTGCCGCGTCTTATCGGGCCTACAAAAGTTCTGAACCGT |  |  |
|  | | |
| Repeat region 1: UTI-2_245U [2136831:2137652] forward | | |
| GTGTTCCCCGCGCCAGCGGGGATAAACCG | TTACGCCATCAGTTGAATATGTGCGCGCCATT | Ec, Ec O114:H49, Mc. |
| GTGTTCCCCGCGCCAGCGGGGATAAACCG | CGCTACTTGCGCCGGGCGCAAAACTTGGCTGA | Ec, Ec O114:H49. |
| GTGTTCCCCGCGCCAGCGGGGATAAACCG | GCTTGCGAATGTGTGCCGTAGAACGTGCGTCG | Ec. |
| GTGTTCCCCGCGCCAGCGGGGATAAACCG | ATTTCGCGCTCTCTGTATCGAATGTCACGAAA | Ec. |
| GTGTTCCCCGCGCCAGCGGGGATAAACCG | ATTCAGAAAATTTAAAAATGTGGGCGCGTGGC | Ec ECC-1470, Ec O139:H28 str. E24377A. |
| GTGTTCCCCGCGCCAGCGGGGATAAACCG | GGGGCGAGGTGATCTTTCAATAACAATACAGC | Ec, Ec O139:H28 str. E24377A. |
| GTGTTCCCCGCGCCAGCGGGGATAAACCT | TGAGATGCCGTTTGCCGTTTTTCTGTTTTTTC | Ec, Ec O114:H49. |
| GTGTTCCCCGCGCCAGCGGGGATAAACCG | TTCTCAGGCTTTACCCACTCTTTTACAGTGTC | Ec, Ec O114:H49, Ec O139:H28 str. E24377A. |
| GTGTTCCCCGCGCCAGCGGGGATAAACCA | GAAGTTCGGCATTGCCCGCAAAGATTTGTCAC | Ec, Ec O114:H49, Ec O139:H28 str. E24377A. |
| GTGTTCCCCGCGCCAGCGGGGATAAACCG | GCGATCTCGCGGAATACACCGACGAGGCGGGC | Ec, Ec ATCC 8739, Ec O104:H21 str. CFSAN002236, Ec O22:H8, Ec O157:H7 str. EC10, Ec H20, Ec O152:H23, Ec O112ab:H8, Ec O39:H21, Ec O9:H10, Sf |
| GTGTTCCCCGCGCCAGCGGGGATAAACCG | TAAGGCCGTCGCCGGATCAGCCTGGCTATGCC | Ec, Ec O22:H8, Ec O157:H7 str. EC10, Ec H20, Ec O152:H23, Ec O112ab:H8, Ec O39:H21, Sf. |
| GAGCTCCCCGCGCCAGCGGGGATAAACCG | TTCTTGCGGGTGTTGCAAATATTCTTCACGTA | Ec, Ec O157:H7 str. EC10, Ec H20, Ec O152:H23, Ec O112ab:H8, Ec O39:H21, Sb, Sf. |
| GAGCTCCCCGCGCCAGCGGGGATAAACCG | GACGCCGCCGCCGCGAAGCCGTTTCCGATGTT | Ec, Ec O22:H8, Ec O157:H7 str. EC10, Ec O112ab:H8, Ec O157:H16, Ec O39:H21, Sf. |
| GAGTTCCCCGCGCCAGCGGGGATAAACCA |  |  |
| Repeat region 2: UTI-2_245U [2163352:2164175] forward | | |
| GTGTTCCCCGCGCCAGCGGGGATAAACCG | ATTAACGCGGCCCGCCTCTGCGTTGGCGTGAA | Ec, Ec K-12, Ec O157:H7 str. EDL933, Ec O157:H7, Ec FAP1, Ec O139:H28 str E24377A |
| GTGTTCCCCGCGCCAGCGGGGATAAACCG | CGCGTTTCTGGAACCGTAATTTGCTGCGGTAT | Ec, Ec K-12, Ec O157:H7 str. EDL933, Ec O157:H7, Ec FAP1, Ec O139:H28 str E24377A |
| GTGTTCCCCGCGCCAGCGGGGATAAACCG | CTATTGGCCTCACTGCCACCGCAGTTAATACT | Ec, Ec O25:H4, Ec O25b:H4-ST131, Ep-783R5, Ep-705R4, Ep-720R5, Ep-569R10. |
| GTGTTCCCCGCGCCAGCGGGGATAAACCG | AATTAAAATCCCCGTTTCTGTTACATGGGCGA | Ec, Ec K-12, Ec O157:H7 str. EDL933, Ec O157:H7, Ec O139:H28 str. E24377A. |
| GTGTTCCCCGCGCCAGCGGGGATAAACCG | AATTTTCTTCATTGCTCAAACAGGCTGCATAAC | Ec, Ec K-12, Ec O157:H7 str. EDL933, Ec O157:H7. |
| GTGTTCCCCGCGCCAGCGGGGATAAACCG | TATGCCTCTTTTAAACGCGCCGCCTTTTTGCC | Ec, Ec K-12, Ec O157:H7 str. EDL933, Ec O157:H7, Ec O8:H8. |
| GTGTTCCCCGCGCCAGCGGGGATAAACCG | ACGAAAATCGCCGCCGGTGTCCTCGCTGATCA | Ec, Ec SCLE84, Ec O8:H8, Ec K-12, O157:H7 str. EDL933, Ec O157:H7, Sf. |
| GTGTTCCCCGCGCCAGCGGGGATAAACCG | GACTCAACCGCGCTTCCCGGCCTCACCACTGC | Ec, Ec SCLE84, Ec O8:H8, Ec K-12, O157:H7 str. EDL933, Ec O157:H7. |
| GTGTTCCCCGCGCCAGCGGGGATAAACCG | GCGAAAAAATACGGCTGGATGCGACAAAAAAA | *No similarities found* |
| GTGTTCCCCGCGCCAGCGGGGATAAACCG | GAGAAGAAGCTCCTGAGCTAACCGTTGAAGAGC | Ec, Ec SCLE84, Ec O8:H8, Ec O139:H28 str. E24377A. |
| GTGTTCCCCGCGCCAGCGGGGATAAACCG | CCAAAGAAGAACAACGAGCCAACTGGTTTCAG | Ec, Ec SCLE84, Ec O8:H8, Ec O91:H21, Ec O104:H4, Ec K-12, Ec O157:H7 str. EDL933, Ec O157:H7, Ec O182:H21, Ec O104:H21 str. CFSAN002236, Ec NCCP15648, Ec O104:H4 str. C227-11, Sf. |
| GTGTTCCCCGCGCCAGCGGGGATAAACCG | GCAATTTGTTGTCCGCGATCCGGTACGCGCGT | Ec, Ec SCLE84, Ec O8:H8, Ec O91:H21, Ec K-12, Ec O157:H7 str. EDL933, Ec O157:H7, Ec O182:H21, Ec O104:H21 str. CFSAN002236, Sf. |
| GTGTTCCCCGCGCCAGCGGGGATAAACCG | CGGCTATGGAATTTATGGAGAAGTTTGGTTTT | Ec O111:H-, Ec O111:NM, Ec O26:H11, Ec O45:H2, Ec O91:H21, Ec O103:H2, Ec O152:H23, Sb, Sf. |
| GTGTTCCCCGCGCCAGCGGGGATAAACCG |  |  |
| Repeat region 3: UTI-2_245U [3297360:3297483] ND probably forward | | |
| CGACCCCCACCATGTCAAGGTGGTGCTCTAACCAACTGAGCTA | CGGACGCAGGATGGTGCGTTCAATTGGACTCGAACCAA | Ec, Ef, Ec O22:H8, S sp. PIB. |
| CGACCCCCACCATGTCAAGGTGGTGCTCTAACCAACTGAGCTA |  |  |
| Repeat region 4: UTI-2_245U [3334842:3334977] ND |  |  |
| GCTTTGCGCGGATCAACCTGTTCTTCTGGTTCCGCATTAG | ACTGTTGCTGTTCCAGCTTGCGCGCTTTGGCACGGGCAATAGCGGCTTCGACGGCG | Ec, Ec O22:H8, Ec O104:H4, Ec O157:H7 str. EC10, Sf, Sd. |
| GCTTTGCGCGGATCGACCTGTTCTTCTGGTTCCGCATTAG |  |  |
| Repeat region 5: UTI-2_245U [4026382:4026473] ND | | |
| CCACCTTTTTTACCTGCTTCAGATGC | GCGCTGCGGGTCATTCTTGAAATTACCCCCGCTGTGCTGT | Ec, Ec O22:H8, Ec O104:H4, Ss. |
| CCACCTTTTTTACCTGCTTCTGATGC |  |  |
| Repeat region 6: UTI-2_245U [4371712:4371856] ND Probably Reverse | | |
| GTAGGTCGGATAAGATGCGCAAGCATCGCATCCGACAATAAGTGCCGGATGC | TGCGAAAATGCCTTATCTGGCCTACAGATTCGATGCGATTC | Ec, Ec O22:H8, Ec O104:H4, Eb. |
| GTAGGTCGGATAAGATGCGCAAGCATCGCATCCGACAATAAGTGCCGAATGC |  |  |
| Repeat region 7: UTI-2_245U [4611294:4611390] ND | | |
| TTGTAGGCCTGATAAGATGCGTCAAGC | ATCGCATCAGGCATTGTGCACCAATTGCCGGATGCGGCACCGG | Ec, Ec O22:H8, Ec O104:H4. |
| TTGTAGGCCTGATAAGACGCGTCAAGC |  |  |
|  | | |
| Repeat region 1: UTI-3_455U [500176:500325] ND | | |
| CGCGTCTTATCAGGCCTACGAGTTCGGTGCTGTGTAGGTCGGATAAGGCGTTCA | TGCCGCATCCGACAATAACAGCATTGCCTGATGCGACGCTTG | Ec, Ec O157:H7. |
| CGCGTCTTATCAGGCCTACGAGTTCAGTGCTGTGTAGGTCGGATAAGGCGTTCA |  |  |
| Repeat region 2: UTI-3_455U [1609015:1609162] forward | | |
| GTTCACTGCCGTACAGGCAGCTTAGAAA | GCACACGTCGAGCTGGTGGGGATTAATGCTGG | Ec, Ec 536, Ec O1:HNT, Ec SMS-3-5, Sf, Eh. |
| GTTCACTGCCGTACAGGCAGCTTAGAAA | TTGCCGCGGATCCTGTCTGCCAATAATGACAA | Ec, Em, Ec O99:H6, Ec O25:H4. |
| GTTCACTGCCGTACAGGCAGATAAAATG |  |  |
| Repeat region 3: UTI-3_455U [3094009:3094135] forward | | |
| TTTGTAGGCCTGATAAGACGCGCCAGCGTCGCATCAGGC | TCCGGGTGCCGGATGCAGCGTGAACGCCTTATCCGGCCTACGGCTCGGA | Ec, Sf, Ss |
| TTTGTAGGCCTGATAAGACGCGCCAGCGTCGCATCAGGC |  |  |
| Repeat region 4: UTI-1_774U [4920535:4920667] ND Possible forward | | |
| ACGCTGCCGCGTCTTATCGGGCCTACAAAAGTTCTGAACCGT | GGGCCGGATAAGGCGTTCACGCCGTATCCGGCATACAGTGCCCGATGCG | Ec, Ec O4:H5, O84:H7, Sf, Eh. |
| ACGCTGCCGCGTCTTATCGGGCCTACAAAAGTTCTGAACCGT |  |  |

**Enterobacteria**

Ec: *Escherichia coli;* Ef: *Escherichia fergusonii*; Em: *Escherichia marmotae;* Eb*: Enterobacteriaceae bacterium;* Eh: *Enterobacter hormaechei;* S: *Shigella;* Sd: *Shigella dysenteriae;* Sf: *Shigella flexneri;* Ss: *Shigella sonnei;* Sb: *Shigella boydii.*

**Viruses**

Ep: *Escherichia* phage vB_EcoM*;* Mc, *Myoviridae sp.*ctitt1.
